# Supplementary material for: Assessment of community efforts to advance network-based prediction of protein–protein interactions
Source: Nat Commun. 2023 Mar 22;14:1582. doi: 10.1038/s41467-023-37079-7 (PMC10033937; doi:10.1038/s41467-023-37079-7)
Supplement: Supplementary file 1 — Supplementary Information [file 41467_2023_37079_MOESM1_ESM.pdf]

Assessment of community efforts to advance network-based prediction of protein-protein interactions

Supplementary Information

Xu-Wen Wang[1], Lorenzo Madeddu[2], Kerstin Spirohn[3,4,5], Leonardo Martini[6], Adriano Fazzone[7], Luca Becchetti[6], Thomas P. Wytock[8], István A. Kovács[8,9], Olivér M. Balogh[9], Bettina Benczik[10,11], Mátyás Pétervári[10], Bence Ágg[10,11], Péter Ferdinandy[10,11] , Loan Vulliard[12,13], Jörg Menche[12,13,14], Stefania Colonnese[15], Manuela Petti[6], Gaetano Scarano[15], Francesca Cuomo[15], Tong Hao[3,4,5], Florent Laval[3,4,5,16,17,18], Luc Willems[16,18], Jean-Claude Twizere[17,18], Marc Vidal[3,4], Michael A. Calderwood[3,4,5], Enrico Petrillo[19,20], Albert-László Barabási[19,21,22], Edwin K. Silverman[1], Joseph Loscalzo[19], Paola Velardi[2], Yang-Yu Liu[1,23]

[1]*Channing Division of Network Medicine, Department of Medicine, Brigham and Women's Hospital and Harvard Medical School, Boston, MA 02115, USA.*

[2]*Translational and Precision Medicine Department Sapienza University of Rome, Rome, Italy.*

[3]*Center for Cancer Systems Biology (CCSB), Dana-Farber Cancer Institute, Boston, MA 02215, USA.*

[4]*Department of Genetics, Blavatnik Institute, Harvard Medical School, Boston, MA 02115, USA.*

[5]*Department of Cancer Biology, Dana-Farber Cancer Institute, Boston, MA 02215, USA.*

[6]*Department of Computer, Control, and Management Engineering "Antonio Rubert", Sapienza University of Rome, Rome, Italy.*

[7]*CENTAI Institute, Turin, Italy.*

[8]*Department of Physics and Astronomy, Northwestern University, Evanston, IL 60208 USA.*

[9]*Northwestern Institute on Complex Systems, Northwestern University, Evanston, IL 60208.*

[10]*Cardiometabolic and MTA-SE System Pharmacology Research Group, Department of Pharmacology and Pharmacotherapy, Semmelweis University, Budapest, Hungary.*

[11]*Pharmahungary Group, 6722 Szeged, Hungary.*

[12]*CeMM Research Center for Molecular Medicine of the Austrian Academy of Sciences, Vienna, Austria.*

[13]*Department of Structural and Computational Biology, Max Perutz Labs, University of Vienna, Vienna, Austria.*

[14]*Faculty of Mathematics, University of Vienna, Vienna, Austria.*

[15]*Department of Information Engineering, Electronics, and Telecommunications (DIET), University of Rome "Sapienza", Rome, Italy.*

[16]*Laboratory of Molecular and Cellular Epigenetics, GIGA Institute, University of Liège, Liège, Belgium.*

[17]*Laboratory of Viral Interactomes, GIGA Institute, University of Liège, Liège, Belgium.*

[18]*TERRA Teaching and Research Centre, University of Liège, Gembloux, Belgium.*

[19]*Department of Medicine, Brigham and Women's Hospital and Harvard Medical School, Boston, MA 02115, USA.*

[20]*Department of General Internal Medicine and Primary Care, Brigham and Women's Hospital, Boston, MA 02115, USA.*

[21]*Network Science Institute and Department of Physics, Northeastern University, Boston, MA 02115, USA.*

[22]*Department of Network and Data Science, Central European University, Budapest H-1051, Hungary.*

[23]*Center for Artificial Intelligence and Modeling, The Carl R. Woese Institute for Genomic Biology, University of Illinois at Urbana-Champaign, Champaign, IL 61801, USA*

(Dated: March 15, 2023)

CONTENTS

I. Categories of PPI prediction methods 4

A. Similarity-based methods 5

B. Probabilistic methods 12

C. Factorization-based methods 14

D. Diffusion-based methods 15

E. Machine Learning method 17

II. Supplementary tables 21

III. Supplementary figures 25

References 39

I. CATEGORIES OF PPI PREDICTION METHODS

Protein-protein interaction (PPI) networks, or the interactome, describe physical interactions between proteins that mediate signaling, regulatory, and transport events in the cell, or assemble into (multi-) protein complexes [1–3]. Interactomes are a specific category of complex network [4–7]. Therefore, link prediction applicable to general complex networks can also be directly applied. In the following, we describe the existing link prediction methods for general networks and some methods specifically designed for PPI prediction.

A network can be mathematically represented by a graph $G(\mathcal{V}, \mathcal{E})$, where $\mathcal{V} = \{1, 2, \ldots, N\}$ is the node set and $\mathcal{E} \subseteq \mathcal{V} \times \mathcal{V}$ is the link set. A link is a node pair (i, j) with $i, j \in \mathcal{V}$, representing a certain interaction, association or physical connection between nodes i and j. Link prediction aims to infer the missing links or predict future links between currently unconnected nodes based on the observed links [8–10].

In this survey, we consider network-based methods, that is, methods that mostly or exclusively leverage local or global connectivity properties of interactomes, rather than biological features, such as protein sequence or three-dimensional structure [11]. One advantage of connectivity-based methods is that some biological features like three-dimensional structure are currently known only for a small percentage of proteins in the interactome, thereby limiting their usage in large-scale PPI prediction, which is the aim of this paper. We grouped the prediction strategies considered in this study, into five classes: similarity-based methods, probabilistic methods, factorization-based methods, diffusion-based methods, and machine learning methods. Similarity methods, traditionally used in the network link prediction literature, exploit the similarity of connectivity patterns of individual nodes in the network. Probabilistic methods define a probability distribution over unobserved links. Factorization-based methods attempt to capture the information of both global structure and clustering structure of a network, using low rank approximations as well as blocks in networks' adjacency matrices. Diffusion methods are stochastic processes, such as random walks, that can be used to extract information about nodes or dense groups of nodes in a network, and to predict interactions. Finally, researchers have explored several machine learning techniques ranging from classic learning methods to deep learning methodologies. Popular deep learning approaches for link prediction rely on novel techniques such as the Graph Neural Networks (GNNs), to exploit complex patterns in network topology.

A. Similarity-based methods

In similarity-based methods, each non-observed node pair (i, j) is assigned a similarity score s_{ij}. A higher score is assumed to represent a greater probability of link existence. The similarity score can be defined in many different ways and may rely on local information only, or some combination of local and global information

Common Neighbors. The common neighbors algorithm quantifies the overlap or similarity of two nodes as follows [12]:

$$s_{ij} = |\Gamma(i) \cap \Gamma(j)|, \tag{1}$$

where $\Gamma(i)$ denotes the set of neighbors of node i, $\cap$ denotes the intersection of two sets and $|X|$ denotes the cardinality or size of set X.

Adamic-Adar Index. This similarity index measures the similarity between two entities based on the connectivity of their shared neighbors [13]:

$$s_{ij} = \sum_{m \in \Gamma(i) \cap \Gamma(j)} \frac{1}{\log|\Gamma(m)|}. \tag{2}$$

Jaccard Index. The Jaccard index measures the overlap of two nodes normalized by their total number of neighbors [14]:

$$s_{ij} = \frac{|\Gamma(i) \cap \Gamma(j)|}{|\Gamma(i) \cup \Gamma(j)|}, \tag{3}$$

where $\cup$ denotes the union of two sets.

Cosine Similarity (i.e. Salton Index). The cosine similarity is closely related to the Jaccard index, with the normalization as the product of the node degrees:

$$s_{ij} = \frac{|\Gamma(i) \cap \Gamma(j)|}{\sqrt{|\Gamma(i)||\Gamma(j)|}}. \tag{4}$$

Preferential Attachment Index. This index assumes that the existent likelihood of a link between two nodes is proportional to the product of their degrees [15]:

$$s_{ij} = k_i k_j, \tag{5}$$

Resource Allocation Index. This index is based on a resource allocation process between pairs of nodes [16, 17]. The similarity between a node pair (i, j) is defined as the amount of resource j received from i through their common neighbors:

$$s_{ij} = \sum_{m \in \Gamma(i) \cap \Gamma(j)} \frac{1}{k_m}. \tag{6}$$

Here, we assume each common neighbor has a unit of resource and will equally distribute among all its neighbors.

Hub Promoted Index. This index is used to measure the link formation between hub nodes and low-degree nodes [18]:

$$s_{ij} = \frac{|\Gamma(i) \cap \Gamma(j)|}{\min(|\Gamma(i)|, |\Gamma(j)|)}. \tag{7}$$

Hub Depressed Index. This index is used to measure the link formation between hub nodes [18]:

$$s_{ij} = \frac{|\Gamma(i) \cap \Gamma(j)|}{\max(|\Gamma(i)|, |\Gamma(j)|)}. \tag{8}$$

Sorensen Index. This index is very similar to Jaccard index but with more robustness against the outliers [19]:

$$s_{ij} = \frac{|\Gamma(i) \cap \Gamma(j)|}{k_i + k_j}. \tag{9}$$

L3 Index. This index is based on the network paths of length three [20]:

$$s_{ij} = \sum_{u,v} \frac{a_{iu} a_{uv} a_{vj}}{\sqrt{k_u k_v}}, \tag{10}$$

where $a_{iu} = 1$ if node i and u interact, and zero otherwise.

Sim Index. This index combines the Jaccard index and the L3 index together [21]:

$$S = AJ + JA, \tag{11}$$

where A is the adjacency matrix and J is the Jaccard similarity matrix.

Katz Index. The Katz index is based on a weighted sum over the collection of all paths connecting nodes i and j [18]:

$$s_{ij} = \sum_{l=1}^{\infty} \beta^l (A^l)_{ij}, \tag{12}$$

where β is a damping factor that gives the shorter paths more weights, and A is the adjacency matrix of the network. The $N \times N$ similarity matrix $S = (s_{ij})$ can be written in compact form as [22]:

$$S = (I - \beta A)^{-1} - I, \tag{13}$$

where I is the identity matrix. The damping factor β is a free parameter and should be less than the reciprocal of the absolute value of the largest eigenvalue $|\lambda_{\max}|$ of A.

Structural Perturbation Method. This method assumes that a group of links is predictable if removing them has only a small effect on the eigenvalues of the network's adjacency matrix, which serve as proxies for the network's more general structural features [23]. This method proceeds by dividing an existing network A into a "training" subset that is used to calculate the eigenvalues and eigenvectors of the remaining network $A^R_{ij} = \sum_{k=1}^{N} x_{ik} x_{kj} \lambda_k$, and a "validation" subset that will be used to characterize the predictability, ΔE, which has size L [23]. The ΔE is projected onto the eigenvectors, $\Delta\lambda_k = \frac{\sum_l \sum_m x_{kl} \Delta E_{lm} x_{mk}}{\sum_l x_{kl} x_{lk}}$, where $\Delta\lambda_k$ is the shift in eigenvalues that best approximates ΔE. The best-fit adjustments are added to the eigenvalues and multiplied by the eigenvectors to obtain a perturbed adjacency matrix: $\tilde{A}_{ij} = \sum_{k=1}^{N} (\lambda_k + \Delta\lambda_k) x_{ik} x_{kj}$. The L largest elements of $\tilde{A}_{ij}$ are selected, such that $A_{ij} \neq 1$, resulting in a set of edges called E^L. The Structural Perturbation Index is then $|E^L \cap \Delta E|/L$.

When applying the structural perturbation method to a given network, a byproduct is

the so-called structural consistency index:

$$\sigma_c = \frac{|E^L \cap \Delta E|}{|\Delta E|}, \tag{14}$$

which can be used to quantify the predictability of the network.

MPS. This method is based on three measures: $\text{MaxSimScore}_{\text{Topological}}$, PAscore, and $\text{MaxSimScore}_{\text{Biological}}$ [8, 21, 24, 25]. More formally, given two proteins u and v, their Jaccard Index $J(u, v)$ is defined as:

$$J(u, v) = \frac{|\Gamma(u) \cap \Gamma(v)|}{|\Gamma(u) \cup \Gamma(v)|}$$

where $\Gamma(u)$ is the set of u's neighbors in the PPI network. The Jaccard Index can be used to identify proteins with similar interfaces, and we can leverage this information to score potential protein interactions.

To better understand how Jaccard Index can be useful in scoring potentially interacting pairs, consider the following scenario: assume we have a protein pair (u, v) for which we want to assess likelihood of interaction. If u is similar to the neighbors of v (i.e., they largely share the same interaction partners) and v is similar to the neighbors of u, then it is more likely for u and v to share complementary binding sites. Thus, they are more likely to interact.

More formally, given proteins u and v, we define their *interaction likelihood* as follows:

$$\text{MaxSimScore}_{\text{Topological}}(u, v) = \max_{x \in \Gamma(v)} J(u, x) + \max_{y \in \Gamma(u)} J(y, v),$$

where $\Gamma(u)$ is the set of neighbors of u. The proposed method is a modification of the `sim` method proposed by Chen et al [21], where the sums are replaced by max functions. PAscore is defined as the product of degrees of a protein pair (u, v), i.e., PAscore $= k_u \times k_v$.

Assuming that proteins that are biologically similar to other interacting proteins are more likely to interact, we designed a framework that leverages proteins' primary sequence and priori knowledge of interacting proteins to identify new candidate interactions. This framework is heavily inspired by the PIPE algorithm [24], a well known protein interaction prediction engine that leverages recurring short polypeptide sequences between known interacting protein pairs to score candidate pairs. In other words, given a candidate pair (u, v) and a known interacting pair (a, b), if multiple regions u_i of u's primary structure are similar to analogous regions in a and the same happens for v's primary structure resemble with respect to b, u and v are more likely to be a interacting protein pair.

Starting from PIPE [24], we designed a simplified framework in which we don't directly consider all co-occurrences of two sub sequence pairs (u_i, v_j), but we use them to define a protein similarity measure. In more detail, we define the biological similarity of two proteins u and a as follows:

$$S(u,a) = \begin{cases} 1, & \text{if } J'(s_u, s_a) > 0 \\ 0, & \text{otherwise} \end{cases} \tag{15}$$

where $J'(s_u, s_a)$ is the Jaccard Index between the set of k-mers of the primary structures of protein u and a. Given a pair of no-interacting proteins (u, v) and the knowledge graph of interacting proteins $G(V, E)$, the algorithm computes the likelihood of the interaction as the following steps:

- **Step 1:** generate A, the set of proteins that are biologically similar to u. More formally, A is defined as follows:

$$A = \left\{ a \in V \middle| S(u,a) = 1 \right\} \tag{16}$$

- **Step 2:** generate R, the set of proteins that interact with at least one protein in A. We can define R as follows:

$$R = \left\{ x \in V \middle| \exists a \in A | (x,a) \in E \right\} \tag{17}$$

- **Step 3:** generate B, the set of proteins that are biologically similar to v. This set is defined the same way we defined A:

$$B = \left\{ b \in V \middle| S(v,b) = 1 \right\} \tag{18}$$

- **Step 4:** compute the likelihood of the interaction (u, v) as the size of intersection between R and B(i.e. $\text{DirSimScore}_{\text{Biological}}(u, v) = |R \cap B|$).

- **Step 5:** compute $\text{DirSimScore}_{\text{Biological}}(v, u)$ by executing, another time, steps from 1 through 4, this time switching u with v.

- **Step 6:** return the following score as a measure of the interaction likelihood of proteins u and v:

$$\text{MaxSimScore}_{\text{Biological}}(u,v) = \max\left(\text{DirSimScore}_{\text{Biological}}(u,v), \text{DirSimScore}_{\text{Biological}}(v,u)\right) \tag{19}$$

For each protein u, our framework pre-computes the set A with a computational cost of $O(P^2L)$ where P is the number of proteins and L is the average protein primary sequence length. This way, steps 1 and 2 have constant time complexity. Finally, it finds R in and computes the size of the intersection in $O(P)$. Thus, the overall time complexity is $O(P^2L)$ that is very small if we compare it with the time complexity of PIPE that is $O(P^3L^2)$.

Finally, we create a framework that leverages both biological and topological information, by aggregating the topological and the biological scores of a candidate pair (u, v) via a convex combination. More formally, we defined the final likelihood of a candidate pair of proteins (u, v) as:

$$\text{MPS(T \& B)}_\beta(u, v) = \beta \cdot \text{MaxSimScore}_{\text{Topological}}(u, v) + (1 - \beta) \cdot \text{MaxSimScore}_{\text{Biological}}(u, v) \tag{20}$$

where $\beta \in \big[0, 1\big]$, and with both scores suitably normalized before the computation of the sum.

We refer to the $\text{MPS(T \& B)}_\beta(u, v)$ method as the **MPS(T)** method, when $\beta = 1$; in a similar fashion, when $\beta = 0.5$, we refer to the $\text{MPS(T \& B)}_\beta(u, v)$ method as the **MPS(T & B)** method. The code for MPS is available at: `https://github.com/spxuw/PPI-Prediction-Project`.

RNM. This method is an extension of the $L3$ method, which has previously been shown to be effective in predicting PPIs [20, 26]. The $L3$ method has no free parameters, and thus makes predictions irrespectively of the amount of incompleteness in the data. In practice, we need less predictions when the measured network is almost complete and more if the dataset is missing most connections. We expect that a method with at least one adjustable parameter can account for the amount of incompleteness and should be able to out-perform the standard $L3$ method.

Our starting point is an edge prediction method of the form

$$\mathbf{P} = \mathbf{X}\mathbf{A}\mathbf{X}^\intercal \tag{21}$$

where A is the $n \times n$ adjacency matrix of the observed PPI network, $\mathbf{X}$ is an $n \times n$ matrix to be determined, and n is the number of proteins in the screen. In the case of $L3$, $\mathbf{X}_{\text{L3}} = \mathbf{A}\mathbf{D}^{-1/2}$, where $\mathbf{D} = \text{diag}(d_i)$, the matrix with the degrees of the nodes of $\mathbf{A}$ on the diagonal. In addition, we consider two alternative methods of the form $\mathbf{X} = |\mathbf{A}|(|\mathbf{A}| + \mathbf{N})^{-1}$, with

$|A| = \sqrt{A^2}$ and $\mathbf{N}_1 = \mathrm{diag}\left(ad_i^{1/c}\right)$ in the first method. In the second method, we first decompose $\mathbf{A} = \mathbf{U}\mathbf{\Lambda}\mathbf{U}^\intercal$, where $\mathbf{\Lambda} = \mathrm{diag}\lambda_i$ with λ_1 being the largest eigenvalue. Then, we consider a method of the form $\mathbf{N}_2 = \mathbf{U}\,\mathrm{diag}(M)\,\mathbf{U}^\intercal$, where $M = b\lambda_1$. Our predictions were made with the parameters $a = 3$, $c = 3$, and $b = 0.95$. Both methods give a small number of new predictions when N is negligible compared to $|A|$, i.e. when a or b is sufficiently small.

Not knowing *a priori* which method is expected to perform best, we choose to use an average of the predictions of the three methods. For the internal cross-validation, we define r_{ij} to be the normalized rank i^{th} interaction predicted by the jth method, with the highest score being $r_{ij} = 1$. Then, we use $\bar{r} = \sum_j r_{ij}/3$ to rank the predictions and evaluate against the test set in terms of the required statistics.

For the external validation, predictions are made for all three methods for each of 3 assays in the human interactome. Within each assay, the predictions are merged and averaged as described in the preceding paragraph, except that the ranks are unnormalized. Since the assays cover largely disjoint sets of genes, the absence of a prediction of a particular pair in one assay can result from that assay's failure to cover the pair in question, or it may reflect a genuine absence of interaction. To merge the predictions of the three assays, we define the rank to be $\bar{r}_{ik}$, where k^{th} is an index over assays. We take the top $10,000$ predicted ranks for each assay and assign $\bar{r}_{ik} = 0$ if the ith pair is queried but missing from these entries in the kth assay, and $\bar{r}_{ik} = \min(R_0, \min_{k\neq l}\bar{r}_{il})$ with $R_0 = 2,500$, otherwise. The final rankings are then $\bar{r}' = \sum_k \bar{r}_{ik}/3$. The treatment of the missing entries prioritizes pairs that appear in multiple assays as these pairs are likely more robust than the top predictions in each assay without allowing the absence of a pair in a given assay to increase its average rank. The code for RNM is available at: `https://github.com/spxuw/PPI-Prediction-Project`.

Other Similarity-Based Methods in the Literature. There are some additional traditional similarity-based methods in the literature, e.g., the Local Leicht-Holme-Newman Index [27], the Individual Attraction Index [28], the Mutual Information [29], the CAR-Based Indices [30], and the Global Leicht-Holme-Newman Index [27], which can also be used to tackle the link prediction problem (see surveys [31–33] for details).

Most of the similarity and diffusion based link prediction methods are available in the MATLAB implementation of SEAL [34]:
`github.com/muhanzhang/SEAL/tree/master/MATLAB`.

B. Probabilistic methods

These methods are based on the concept of maximum likelihood, assuming that real networks have some structure, i.e., hierarchical or community structure. The goal of these algorithms is to select model parameters that can maximize the likelihood of the observed structure.

Stochastic Block Model. As one of the most general network models, the stochastic block model (SBM) assumes that nodes are partitioned into groups, and the probability that two nodes are connected depends solely on the groups to which belong [35, 36]. The SBM assumes that a link with higher reliability has higher existent probability, and the reliability of a link is defined as [37]:

$$R_{ij}^{L} = \frac{1}{Z} \sum_{P \in \mathcal{P}} \left(\frac{l_{\sigma_i \sigma_j}^{O} + 1}{r_{\sigma_i \sigma_j} + 2} \right) \exp[-\mathcal{H}(P)], \tag{22}$$

where $\mathcal{P}$ represents the partition space of all possible partitions, σ_i is the group to which that node i belongs in the partition P, $l_{\sigma_i \sigma_j}^{O}$ is the number of links between groups σ_i and σ_j in the observed network, $r_{\sigma_i \sigma_j}$ is the maximum possible number of links between them, and the function $\mathcal{H}(P) \equiv \sum_{\alpha \leq \beta} [\ln(r_{\alpha\beta} + 1) + \ln \begin{pmatrix} r_{\alpha\beta} \\ l_{\alpha\beta}^{O} \end{pmatrix}]$, and $Z \equiv \sum_{P \in \mathcal{P}} \exp[-\mathcal{H}(P)]$. In practice, we can use the Metropolis algorithm to sample relevant partitions that significantly contribute to the sum over the partition space $\mathcal{P}$. This approach allows us to calculate the link reliability efficiently.

Hierarchical Structure Model. Many real networks have hierarchical structure, which can be represented by a dendrogram D. One can assign a probability p_r to each internal node r of D with the connecting probability of a pair of leaves given by $p_{r'}$, where r' is the lowest common ancestor of these two leaves. Denote E_r as the number of edges in the network whose endpoints have r as their lowest common ancestor in the dendrogram D. Let L_r and R_r be the number of leaves in the left and right subtrees rooted at r, respectively. The likelihood of D associated with a set of probabilities $\{p_r\}$ is, then, given by [38]:

$$\mathcal{L}(D, \{p_r\}) = \prod_{r \in D} p_r^{E_r} (1 - p_r)^{L_r R_r - E_r}. \tag{23}$$

For a specific D, the probabilities $\{\overline{p}_r\}$ that maximize $\mathcal{L}(D, \{p_r\})$ are edges between the two subtrees of r that are present in the network:

$$\{\overline{p}_r\} = \frac{E_r}{L_r R_r}. \tag{24}$$

Evaluating the likelihood $\mathcal{L}(D, \{p_r\})$ at this maximum yields

$$\mathcal{L}(D) = \prod_{r \in D} \left[\bar{p}_r^{\bar{p}_r} (1 - \bar{p}_r)^{1 - \bar{p}_r} \right]^{L_r R_r}. \tag{25}$$

One can use the Markov chain Monte Carlo (MCMC) method to sample a large number of dendrograms D with probability proportional to their likelihood $\mathcal{L}(D)$. For each pair of unconnected leaves i and j, we calculate the connection probability p_{ij} for each D, and then calculate the average $\langle p_{ij} \rangle$ over all the sampled dendrograms. The $\langle p_{ij} \rangle$ value yields the existence probability of the link between nodes i and j. For each non-existent link or node pair (i, j), we calculate the average connecting probability $\langle p_{ij} \rangle$ over all sampled dendrograms, with node pairs with highest $\langle p_{ij} \rangle$ being missing links.

RepGSP. This method is based on the Graph Signal Processing (GSP) technique [39]. The method consists of a Multiscale Markov Random Field (MRF) of the interactome. MRF is a Markov technique modeling the network's edges based on features associated with the nodes [40]. In order to capture latent information of the network, signal of graphs (SoGs) are exploited. The SoGs are designed to capture the topological patterns of the network by resorting to a Markovian model taking into account either the pathways of length 3 between two nodes (i.e., proteins) and the community structure of the graph. The method is completely unsupervised and it is based only on the structural information of the network. In particular it weights attractive or repulsive behaviour of graph nodes belonging to the same community [41]: herein, RepGSP rewards links between "repulsive nodes" (i.e., nodes belonging to different communities).

Other Probabilistic Models in the Literature. There are also many probabilistic model based link prediction methods (which typically have high time complexity [42]), such as the Probabilistic Relational Model [43], the Probabilistic Entity Relationship Model [44], and the Stochastic Relational Model [45], as well as many other methods.

C. Factorization-based methods

These methods posit that the observed network structure has can be generated from a lower-dimensional *latent space* and use matrix factorization or completion techniques to find a mapping to embed the original network, into a lower dimension such that the similar nodes in the original network tend to have similar latent representation features.

Matrix Factorization. Matrix factorization aims to decompose the observed adjacency matrix or node attribute data into two or more matrices through supervised and unsupervised approaches. Denote a data matrix X, with p rows and n columns. Each column represents a sample and each row represents a particular feature. The data matrix can be factorized as [32, 46]:

$$X \approx FG^T, \tag{26}$$

where $X \in \mathbb{R}^{p\times n}$, $F \in \mathbb{R}^{p\times k}$, and $G \in \mathbb{R}^{n\times k}$. Matrix F represents the bases of the latent space, and matrix G contains combinations of coefficients of the bases and k is a parameter that specifies the dimension of latent space ($k < n$). The matrix factorization methods can be categorized by the sign constraints on the above three matrices, which are denoted by a subscript. For example, singular value decomposition (SVD): $X_\pm \approx F_\pm G_\pm^T$; Non-negative matrix factorization (NMF): $X_+ \approx F_+ G_+^T$; Semi-NMF: $X_\pm \approx F_\pm G_+^T$. Convex-NMF: $X_\pm \approx F_\pm W_+ G_\pm^T$'. Solving the factorization problem can be modeled as a constrained and potentially regularized least squares optimization problem.

Low-Rank Matrix Completion. The goal of matrix completion is to recover a low-rank matrix L from a large matrix A, which can be used to infer the missing links of a network. The matrix L can be calculated by solving the convex optimization problem [47]:

$$\min_{L,S} \|L\|_* + \lambda |S|_1, \text{s.t. } A = L + S. \tag{27}$$

Here, S is a sparse matrix. $\|\cdot\|_*$ denotes the nuclear norm (sum of singular values) of a matrix, and $|\cdot|_1$ represents the sum of the absolute values of each matrix element and λ is a positive parameter. The matrix S is defined as follows: if two nodes (i, j) are connected in the observed network, then the score $S_{ij} = A_{ij}$; otherwise, $S_{ij} = L_{ij}$.

GLEE. The Geometric Laplacian Eigenmap Embedding (GLEE) method uses the Laplacian matrix to find the embedding using (simplex) geometric properties, rather than spectral properties, through leveraging the simplex geometry of the network [48]. The Geometric Laplacian Eigenmap Embedding is available at: `github.com/leotrs/glee`. We used the default parameters in GLEE and dimensionality is set as $d = 128$.

D. Diffusion-based methods

For a given network, a diffusion process captures the latent structure in the spread of information over the connections. For example, random walks propagate information by sampling network paths following a stochastic process. Diffusion techniques are used in a variety of settings and are integrated with similarity measures or deep learning methods like the Skip-Gram model [49]. Here, we briefly introduce some widely used diffusion-based algorithms.

Average Commute Time. The average commute time index is motivated by a random walk process on the network [7]:

$$s_{ij} = \frac{1}{l^+_{ii} + l^+_{jj} - 2l^+_{ij}}, \tag{28}$$

where l^+_{ij} is the entry of the pseudoinverse of the Laplacian matrix $L \equiv D - A$, where $D = \mathrm{diag}\{k_1, k_2, \cdots, k_N\}$ is the degree matrix and A is the adjacency matrix.

RWR. This method involves a similarity measure based on a random walk with restart sampling. Random walks with restart are random walks with a restart probability [50].

SimRank This method involves a network propagation of node structural context similarity based on object-to-object relationships [51]. SinRank measures similarity of the structural context in which objects occur, based on their relationships with other objects.

Deepwalk. DeepWalk uses local information obtained from truncated random walks to learn latent representations by treating walks as the equivalent of sentences, consisting of two main components: random walk sampling and embedidng learning [52]. Once the

random walk sequence are sampled, these are fed to a Skip-Gram neural network to learn node embeddings [53].

LINE. LINE is a novel network embedding method, with a carefully designed objective function that preserves both the first-order and second-order proximities, which is scale to large directed, undirected and weighted networks [54].

Node2vec. node2vec learns a mapping of nodes to a low-dimensional space of features that maximizes the likelihood of preserving network neighborhoods of nodes. By choosing an appropriate notion of a neighborhood, node2vec can learn representations that organize nodes based on their network roles and/or communities to which they belong [55].

RW2. This method is divided into two steps: representation learning and classification. In the representation learning step, RW2 is applied to the interactome to generate node embeddings. Since RW2 needs categorical features associated with nodes, each node is enriched by the following labels, node ID and biological properties expressed in terms of GO-Terms (biological functions, biological processes and cellular locations). In the classification step, an XGboost method is trained with the embeddings generated by RW2. The method is supervised and is based on structural and biological information in the network (Level-2).

DNN+node2vec. This method consists of two steps. First, it employs node2vec to learn node embeddings [55]. Next, the node embeddings are fed to a deep neural network for PPI prediction. The deep neural network is invariant to the node order in input. The code of DNN+node2vec is available at: `https://github.com/spxuw/PPI-Prediction-Project`.

E. Machine Learning method

Machine learning (ML) is a growing field based on learning techniques to extract regular patterns in the given data. Deep learning (DL) is a sub-field of Machine Learning composed of multi-layered neural network methods. Here, we introduce classic machine learning and deep neural network architectures.

Coding Proteins and K-Nearest Neighbors (Code4+KNN). This method first represents each protein sequence as a vector by using local protein sequence descriptors, and then concatenates the information of the two proteins in the pair into a new pairwise feature vector using four "coding" functions. Finally, a KNN method is trained using the pairwise feature vectors as input [56].

Auto Covariance and Support Vector Machine (AC+SVM). This method is a sequence-based method that combines the auto covariance (AC) measure with the support vector machine (SVM) classifier. AC accounts for the interactions between residues of a certain separation distance in the sequence in an attempt to account for local interactions between amino acids. [57].

Local Protein Sequence Descriptors and Support Vector Machine. This approach combines local protein sequence descriptors with the SVM classifier. Local descriptors account for the interactions between sequentially distant but spatially close amino acid residues in an attempt to capture multiple overlapping continuous and discontinuous binding patterns within a protein sequence [58].

Multi-Scale Continuous and Discontinuous feature Representation and Support Vector Machine (MCD+SVM). This approach uses a novel Multi-scale Continuous and Discontinuous (MCD) feature representation and Support Vector Machine (SVM). The MCD representation gives adequate consideration to the interactions between sequentially distant but spatially close amino acid residues; thus, it can sufficiently capture multiple overlapping continuous and discontinuous binding patterns within a protein sequence. An effective feature selection method mRMR was employed to construct an optimized and more

discriminative feature set by excluding redundant features. Finally, a prediction method is trained and tested using the SVM classifier to predict the interaction probability of protein pairs [59].

Deep-Forest (GcForest). This is a deep-forest-based method for PPIs prediction. First, pseudo amino acid composition (PAAC: an encoding of the amino acids based on their functional role), autocorrelation descriptor (Auto: a measure of how frequently specific amino acids recur), multivariate mutual information (MMI: a method for organizing high-dimensional mutual information terms by the number of variables considered so that the mutual information can be approximated), composition-transition-distribution (CTD. Composition: frequency of amino acids; Transition: frequency of amino acid pairs; distribution: frequency of amino acids in a subdomain in a protein), amino acid composition PSSM (AAC-PSSM: an amino acid position specific substitution matrix constructed from phylogenetic data that measures how frequently the amino acids are substituted in functionally similar genes), and dipeptide composition PSSM (DPC-PSSM: is similar to PSSM, but it is constructed specifically for pairs of amino acids.) are adopted to extract and construct the pattern of PPIs. Secondly, elastic net is used to optimize the initial feature vectors and boost the predictive performance. Finally, the GcForest-PPI method based on deep forest is constructed [60].

FCTP-WSRC. Initially, combinations of the F-vector (a fixed length feature vector containing key information), composition (C) and transition (T) are used to map each protein sequence into numeric feature vectors. Principal component analysis (PCA) is employed to reconstruct the most discriminative feature subspaces, which are subsequently used as input in a weighted sparse representation based classification (WSRC) for prediction [61].

PCA-EELM. This method involves a novel hierarchical principal component analysis-ensemble extreme learning machine (PCA-EELM) model to predict protein-protein interactions using the information contained in protein primary amino acids sequences. In the proposed method, 11,188 PPIs retrieved from the DIP database were encoded into feature vectors by using four kinds of protein sequences descriptors. Next, the PCA method was employed to construct the most discriminative new feature set. Finally, multiple extreme

learning machines were trained and then aggregated into a consensus classifier by majority voting [62].

DeepPPI. This method, called DeepPPI (Deep neural networks for Protein-Protein Interactions prediction), employs deep neural networks to learn effectively the representations of proteins from common protein descriptors [63].

iPPI. First, iPPI proposes the amino acid properties of PPI closely related protein sequences and the influence of an imbalanced training set on prediction accuracy. Next, it encodes the primary sequence with the properties of 20 amino acids and employs a random undersampling strategy to solve the imbalance issue. In the training phase, it builds a hybrid deep neural network to model the encoded sequence profiles of the bootstrapping-based training datasets [64].

DPPI. This method applies a deep, twin-like convolutional neural network combined with random projection and data augmentation to predict PPIs, leveraging existing high-quality experimental PPI data and evolutionary information of a protein pair under prediction [65].

Deep generative model (DGM). The key princple of DGM is to represent the adjacency matrix of a network as an image, then learn hierarchical feature representations of the image by training a deep generative model. Those features correspond to structural patterns in the network at different scales, from small subgraphs to mesoscopic communities. This method does not rely on any domain-specific heuristic and works for general undirected or directed complex networks [66].

cGAN. This method, as described by Balogh et al. [67] consists of training a conditional Generative Adversarial Network (cGAN) method that uses Wasserstein distance-based loss with gradient penalty [68–70]. The method is divided into two steps: representation learning and adversarial learning. In the representation learning step, the authors generate node embeddings by applying node2Vec [55] to the interactome. Next, in the adversarial learning step, they train the cGAN by feeding the node embeddings and adjacency matrices of the subgraphs (generated starting from each node of the interactome) to the cGAN's generator.

In the meanwhile, they feed the same matrices given to the generator and the confidence matrices produced by the generator to the cGAN's discriminator that serves as a critic, scoring the performance of the generator. The cGAN architecture does not perform classification directly for the purpose of link prediction, rather to provide feedback for the training of the cGAN's generator. Thus, the generator (an unsupervised method) can be trained using the loss of the discriminator (a supervised method). A further refined version, titled cGAN2, was also evaluated that uses only the adjacency-based topological information without the node embedding part. The code of cGAN used to generate the presented results is available at: `https://github.com/spxuw/PPI-Prediction-Project` , and a continuously updated version is available at: `https://github.com/semmelweis-pharmacology/ppi_pred`.

GraphSAGE. GraphSAGE is a popular graph representation learning method that uses node feature information to generate node embeddings for previously unseen data. Unlike most of embedding methods that require all nodes in the graph to be present, GraphSAGE trains individual embeddings for each node through learning a function that generates embeddings by sampling and aggregating features from a node's local neighborhood [71].

SEAL. SEAL is a representative GCN-based link prediction method, which combines graph structural features, latent features, and explicit features into a single GCN [34]. In particular, the input to GCN is a local subgraph around each target link. Those local subgraphs capture graph structure feature related to link existence. The latent and explicit features can be naturally combined in GCN by concatenating node embedding and node attributes in the node information matrix for each subgraph. We used the the code found at: `github.com/muhanzhang/SEAL/tree/master/Python`. The parameters used for SEAL are number of hop $h = 1$, the maximum node per hop is 50, the maximum training links is 10,000 for HuRI to reduce the memory requirement, while the default parameters are used for the other 4 organism-specific interatomes. The embedding features of each node were obtained by node2vec: `https://github.com/eliorc/node2vec` with default parameters. The dimensionality of HuRI is $d = 4$ and $d = 128$ for other 4 interactomes. The node attributes of each interactome are obtained from the approach proposed in Ref. [61]; this study developed a method, called FCTP-WSRC, to predict PPIs. Here, we used the first 30 dimensions in PCA analysis as the node attributes of each protein. The sequences were

downloaded from the Uniprot [72].

CGNN. CGNN uses a variational auto-encoder procedure designed to obtain node embeddings using neural networks [73]. The observed variable A, evidence feature X and the latent variable Z are connected by the joint distribution representing the probability of A and X with a given Z. The neural network is used to learn the joint probability via MCMC through supervised learning.

SkipGNN. SkipGNN a graph neural network approach for the prediction of molecular interactions [74]. SkipGNN predicts molecular interactions by not only aggregating information from direct interactions but also from second-order interactions, which the authors call skip similarity. SkipGNN receives neural messages from two-hop neighbors as well as direct neighbors in the interaction network, and non-linearly transforms the messages to obtain useful information for prediction. To inject skip similarity into a GNN, SkipGNN constructs a modified version of the original network, called the skip graph. The SkipGNN is available at: `https://github.com/kexinhuang12345/SkipGNN`. The embedding features of each node were obtained by node2vec: `https://github.com/eliorc/node2vec` with default parameters. The dimensionality is $d = 128$ for all 5 interactomes.

II. SUPPLEMENTARY TABLES

Network	N	E	$\langle k \rangle$	d	l	C	ρ	N_{cc}	Tech
A. thaliana	2,774	6,205	4.474	15	-	0.049	0.00161	120	Y2H
C. elegans	2,528	3,864	3.057	14	-	0.019	0.00121	147	Y2H
S. cerevisiae	2,018	2,930	2.904	14	-	0.0462	0.00144	185	Y2H
H. sapiens (HuRI)	8,272	52,548	12.705	12	-	0.059	0.00154	72	Y2H
H. sapiens (STRING)	6,926	41,948	13.614	21	-	0.477	0.002	308	Hybrid
H. sapiens (BioGRID)	19,665	713,793	72.595	7	-	0.118	0.004	1	Hybrid
A. thaliana LCC	2,532	6,037	4.768	15	4.657	0.051	0.00188	1	
C. elegans LCC	2,214	3,659	3.305	14	5.321	0.019	0.00149	1	
S. cerevisiae LCC	1,647	2,682	3.257	14	5.612	0.057	0.00198	1	
H. sapiens (HuRI) LCC	8,149	52,463	12.876	12	3.844	0.060	0.00158	1	
H. sapiens (STRING) LCC	6,036	41,088	13.614	21	5.117	0.477	0.00226	1	
H. sapiens (BioGRID) LCC	19,665	71,3793	72.595	7	2.799	0.1178	0.00369	1	

Supplementary Table I. Network statistics summary. LCC: largest connected component. N: number of nodes. E: number of edge. $\langle k \rangle$: average degree. d: diameter. l: characteristic path length. Note that l cannot be calculated for disconnected network. For these cases we use symbol $-$ as value for l. C: clustering coefficient. ρ: edge density. N_{cc}: number of connected components. We used the software Cytoscape to calculate these statistics [75].

Method	ρ_0	l_0	f_{sl}
MPS(B&T)	0.1458	2.491	0%
MPS(T)	0.0908	2.718	0%
RNM	0.0943	2.348	0%
SBM	0.0994	1.964	0%
cGAN	0.0238	2.595	99.4%
SEAL	0.0127	3.671	0%
DNN+node2vec	0.0128	3.394	0%

Supplementary Table II. Network features of the positive PPIs predicted by the top-seven methods. Prior edge density ρ_0 measures the edge density of the interactome subgraph induced by the proteins involved in the positive PPIs (among the top-500 predicted PPIs) predicted by a method. Prior average shortest path length l_0 is the average shortest path length between the proteins involved in the positive PPIs (among the top-500 predicted PPIs) predicted by a method. Both ρ_0 and l_0 were calculated in the original interactome (without considering the previously uncharacterized PPIs). f_{sl} represents the fraction of self-loops among the positive PPIs predicted by a method. Note that all the measures presented in this table are based on the positive PPIs within the top-500 predicted PPIs of each method.

Method	Positive count	Negative count	Unsuccessful count	Precision
MPS(B&T)	376	54	70	0.874
MPS(T)	272	86	142	0.759
RNM	274	120	106	0.695
SBM	225	167	108	0.574
cGAN	175	164	161	0.516
SEAL	19	332	149	0.054
DNN+node2vec	10	276	214	0.035

Supplementary Table III. Experimental evaluation of the top-seven human PPI prediction methods. Precision is defined as: Precision=Positive count/(Positive count+Negative count).

III. SUPPLEMENTARY FIGURES

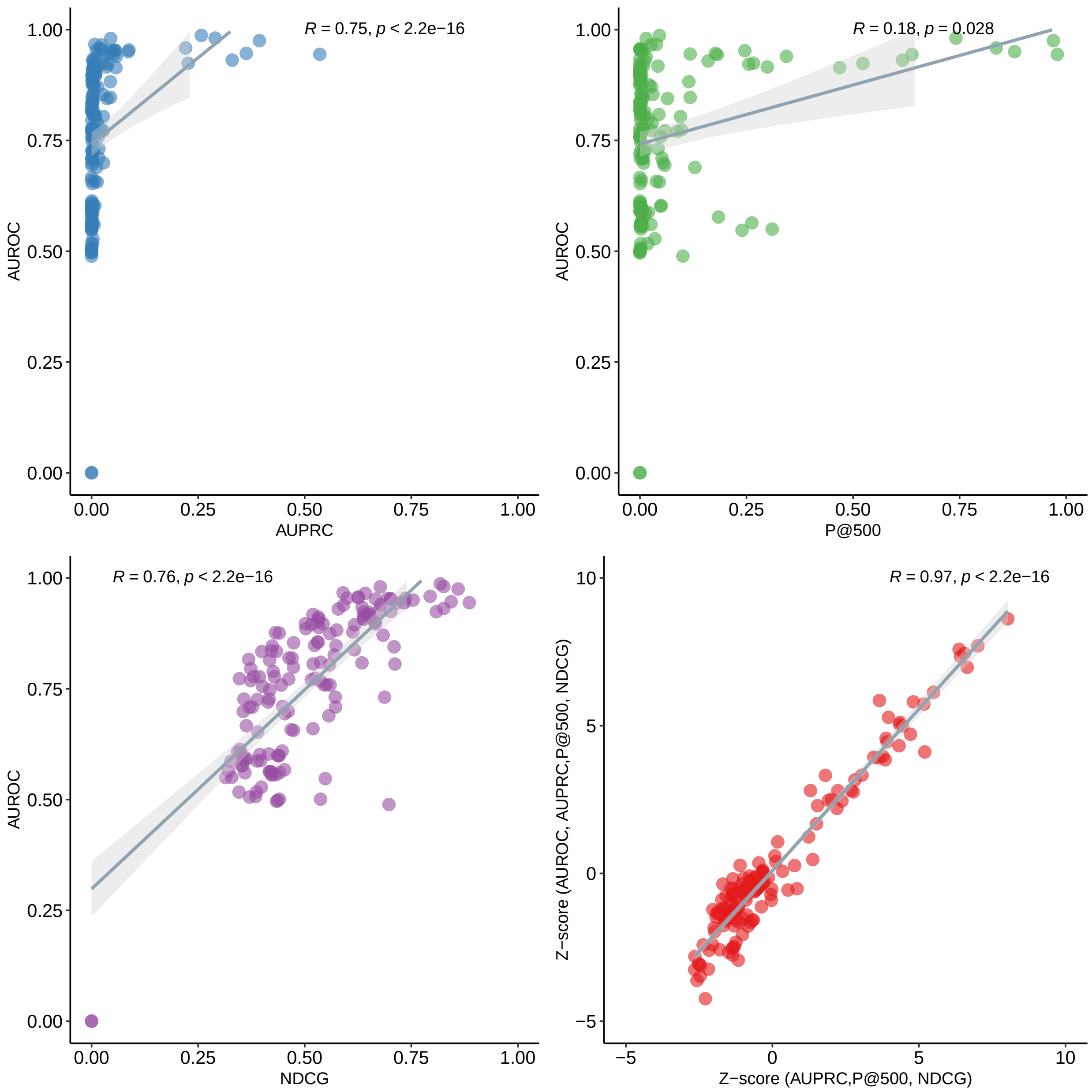

Supplementary Figure 1. **Relationship between AUROC and other performance metrics.** Spearman correlation between AUROC and AUPRC (**a**), P@500 (**b**), and NDCG (**c**). **d:** Pearson correlation between Z-scores including AUROC and excluding AUROC. P values were calculated from two-sided t-test.

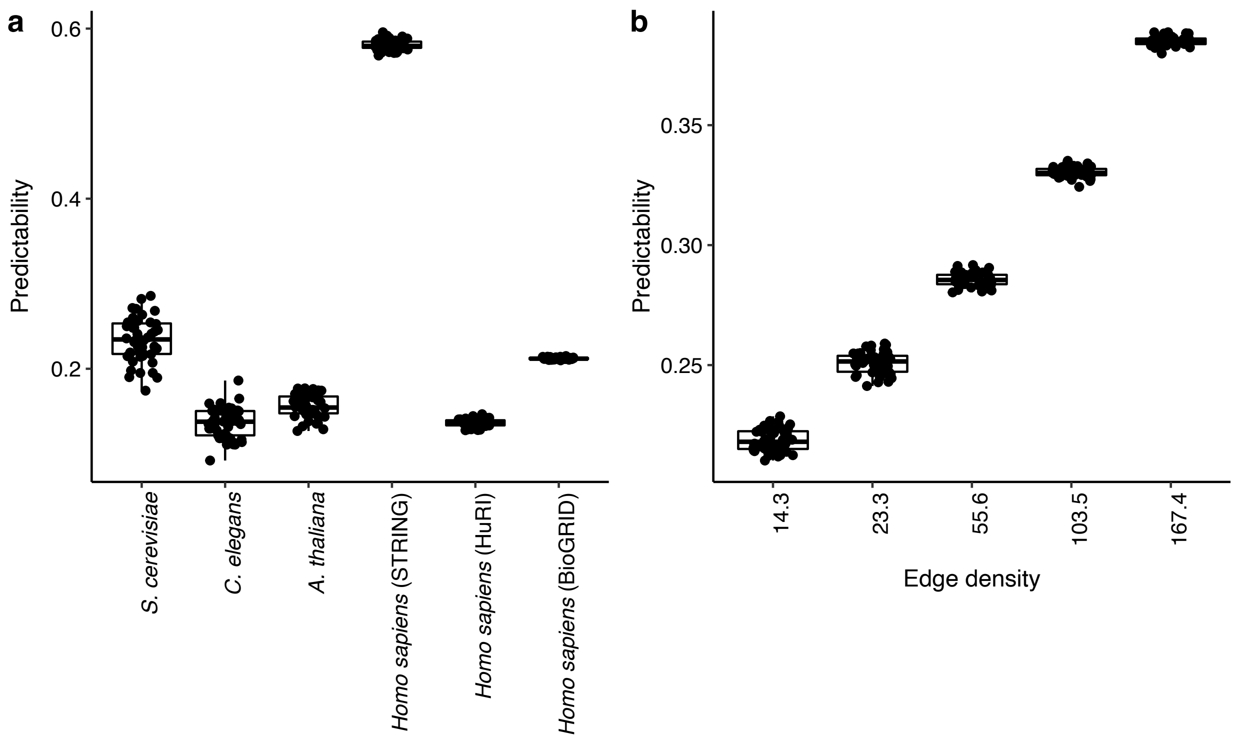

Supplementary Figure 2. **Predictability of different interactomes.** Boxplot shows the predictability over 50 different realizations. For each realization, we randomly split the links into the training set (90%), with the remaining 10% as the test set. To quantify the predictability of each interactome, we calculated its structural consistency index σ_c based on the first-order perturbation of the interactome's adjacency matrix, using the Matlab implementation of the Structural Perturbation Method (SPM) for link prediction [76]. (Note that here we explicitly considered self-loops in the calculations of σ_{c}.) See I A for details on SPM. Boxes indicate the interquartile range between the first and third quartiles with the central mark inside each box indicating the median. Whiskers extend to the lowest and highest values within 1.5 times the interquartile range. **a:** Predictability of real interactomes. **b:** Predictability of synthetic interactomes with different edge density. Here, we generated synthetic interactomes using duplication-mutation-complementation model [77]. Size of the synthetic interactome is 5,000 with a tuning divergence parameter.

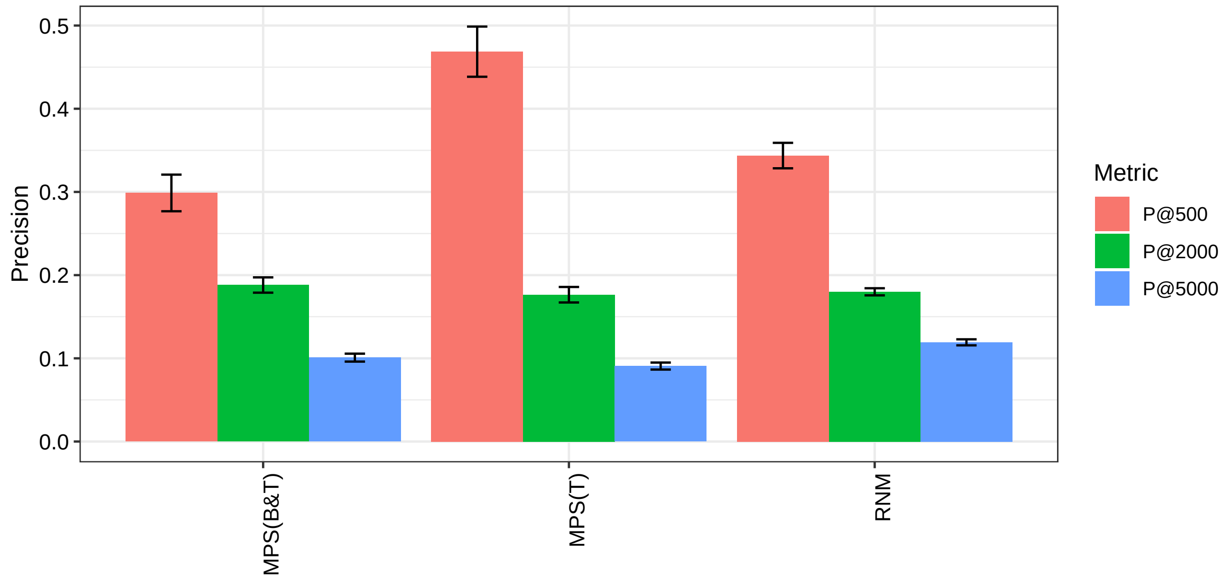

Supplementary Figure 3. **P@K of three top methods: MPS(B&T), MPS(T) and RNM.** Error bar represents the standard deviation among ten realizations of HuRI.

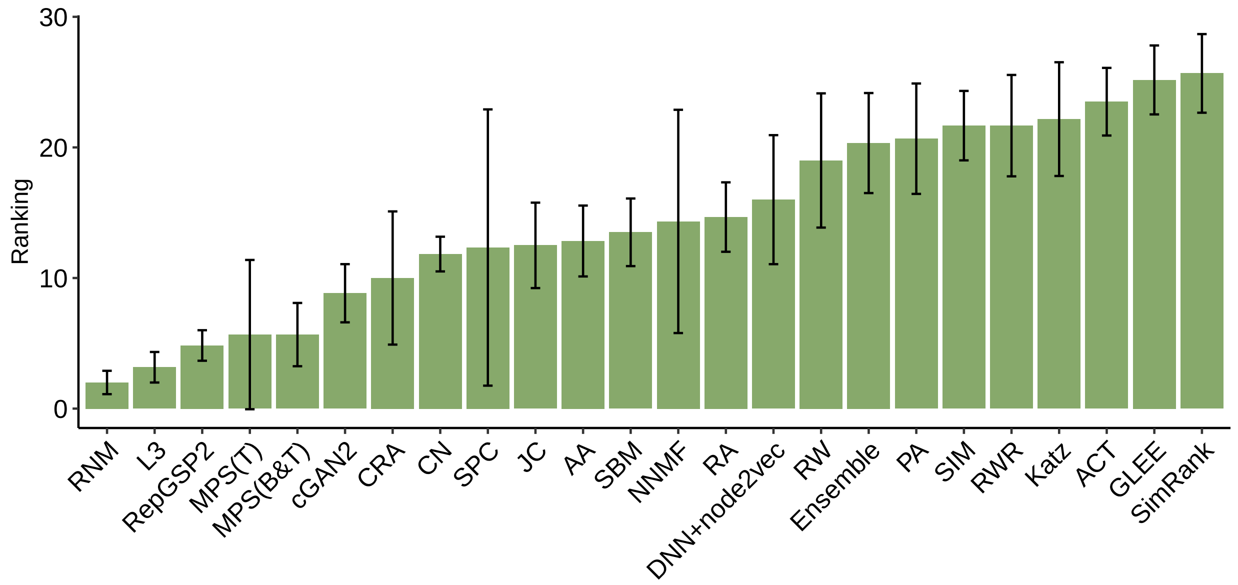

Supplementary Figure 4. **Ranking and variability of the PPI prediction methods over six interactomes.** Bar represents the mean ranking of each method over six interactomes, and the error bar represents the variability of a method (computed as the standard derivation of rankings over six interactomes analyzed in this project). We did not show the variabilities of two methods that were not applied to all the six interactomes.

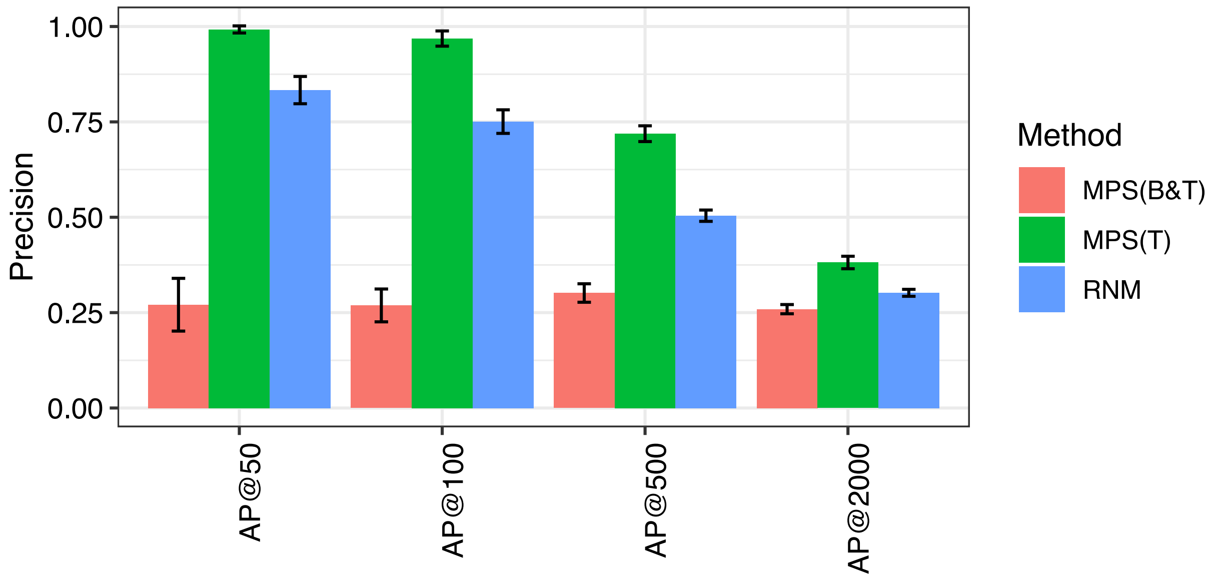

Supplementary Figure 5. **AP@K of three top methods: MPS(B&T), MPS(T) and RNM.** AP@K ($K = 50, 100, 500, 2000$) is computed as the average precision P@i, $i = 1, \ldots, K$. Error bar represents the standard deviation among ten realizations of HuRI.

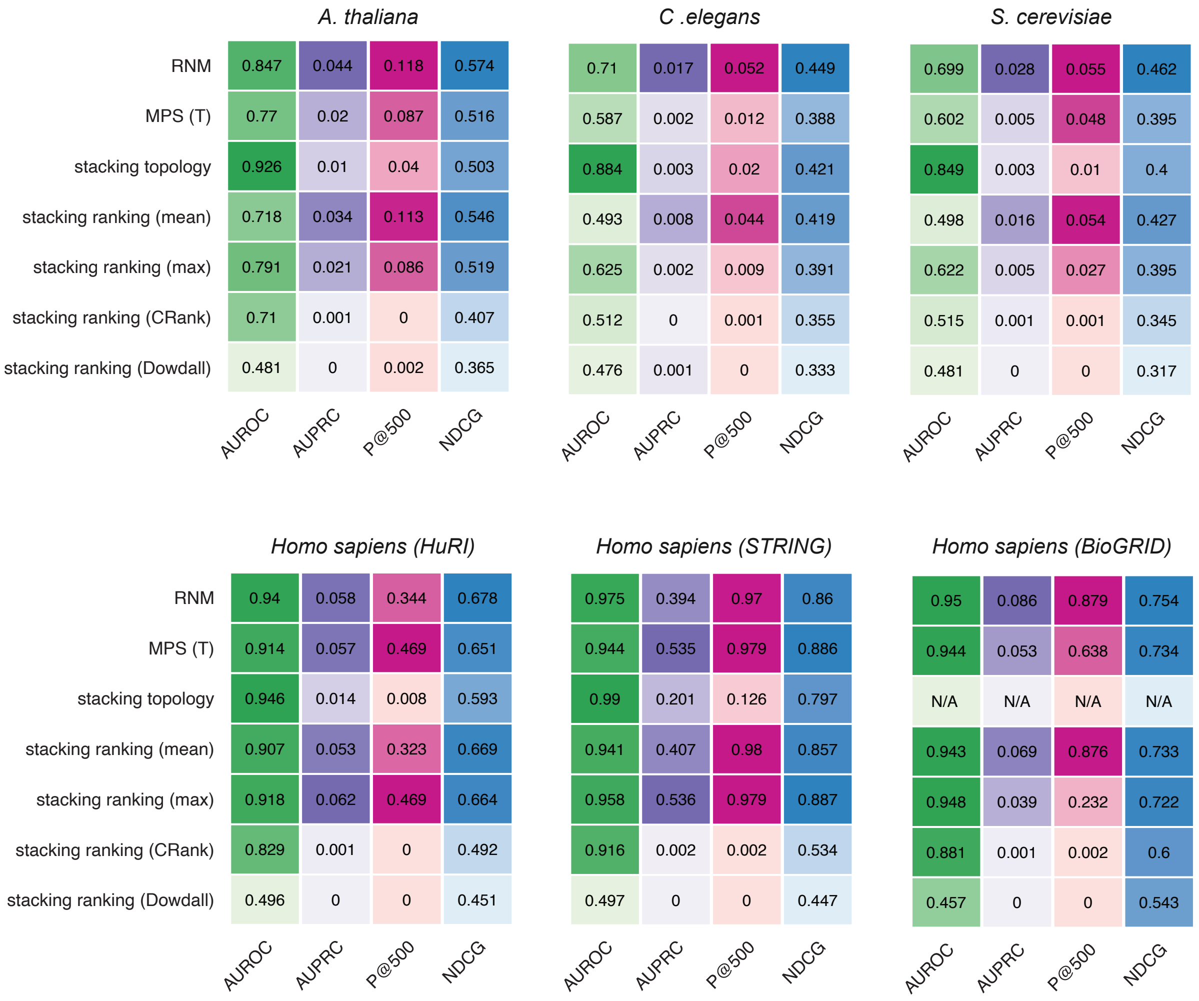

Supplementary Figure 6. **Stacking models do not outperform individual methods in any of the five different interactomes.** Stacking topology (Supervised): we stacked 36 individual topological predictors into a single algorithm, then train a supervised classifier to predict the missing links. Stacking ranking (mean) (Unsupervised): we merged the ranking scores of each link from RNM and MPS, into a single value by taking the mean of the two. Stacking ranking (max) (Unsupervised): we merged the ranking scores of each link from RNM and MPS, into a single value by taking the maximum of two. Stacking ranking (CRank): ranking aggregation using CRank algorithm [78]. Stacking ranking (Dowdall): ranking aggregation using Dowdall method [79]. Note that, the performances of stacking topology on the BioGRID database was not evaluated due to the prohibitive computational cost. We marked its performance as N/A.

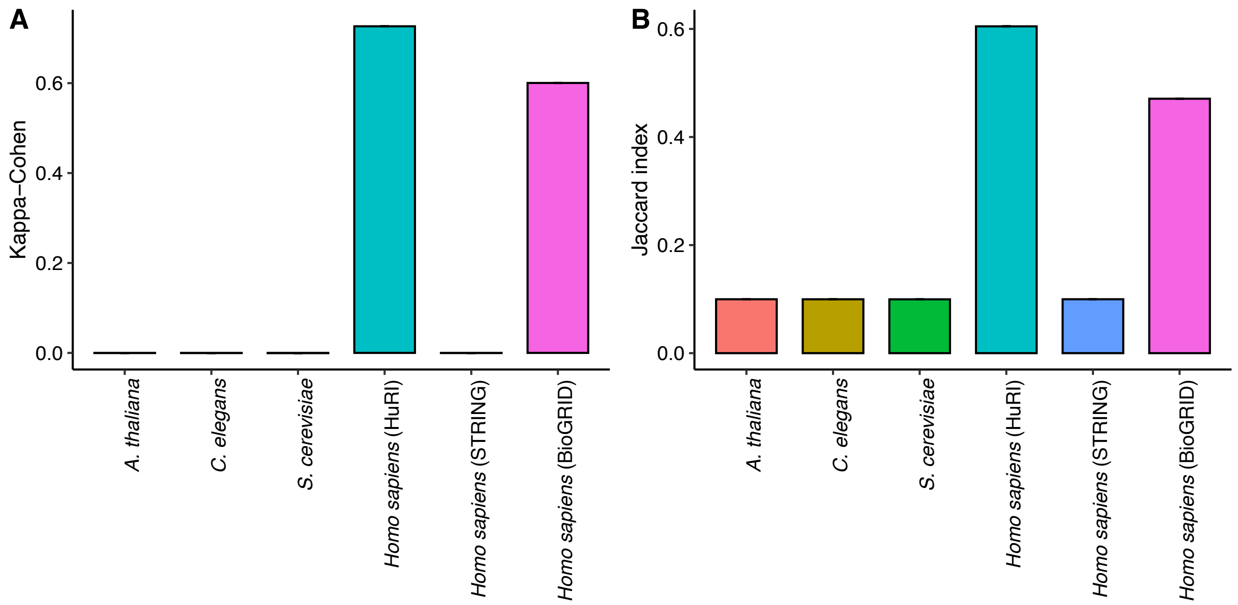

Supplementary Figure 7. **Consistency between the ranking from different methods. a:** Cohen's kappa coefficient between the ranking scores of all test links under 10-fold cross validation by RNM and MPS(T). **b:** The Jaccard index between the ranking scores of RNM and MPS(T). In the calculation of Cohen's kappa coefficient and the Jaccard index, we set the threshold i.e., the fraction of test links, to be 10%, to dichotomize the ranking scores. Boxes indicate the interquartile range between the first and third quartiles with the central mark inside each box indicating the median. Whiskers extend to the lowest and highest values within 1.5 times the interquartile range.

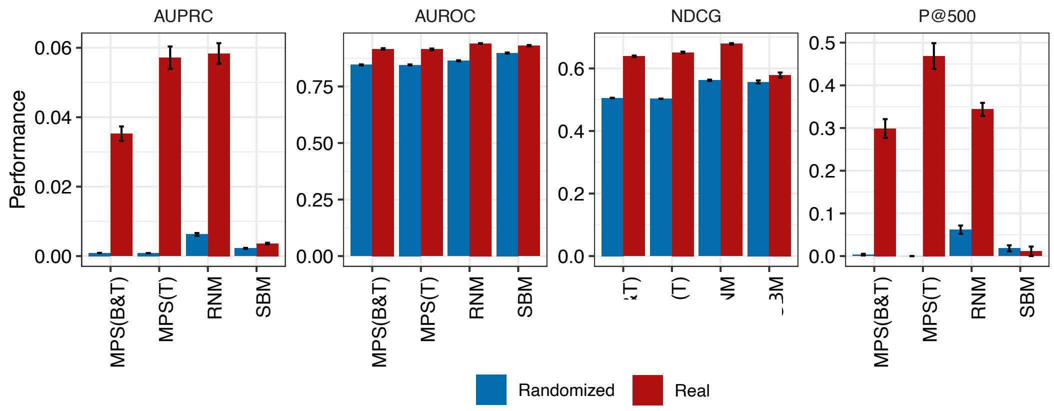

Supplementary Figure 8. **Performance of PPI prediction methods on the degree-preserving randomized interactomes.** We randomly rewired the PPIs of HuRI in $10N$ trials while preserving the original interactome's degree distribution using functions rewire and keeping_degreeseq functions of igraph [80] package. Four prediction methods RNM, SBM, MPS(T), and MPS(B&T) that tend to predict PPIs involving proteins of high degrees were evaluated in the randomized interactome. Error bar represents the standard deviation among 10-fold validations.

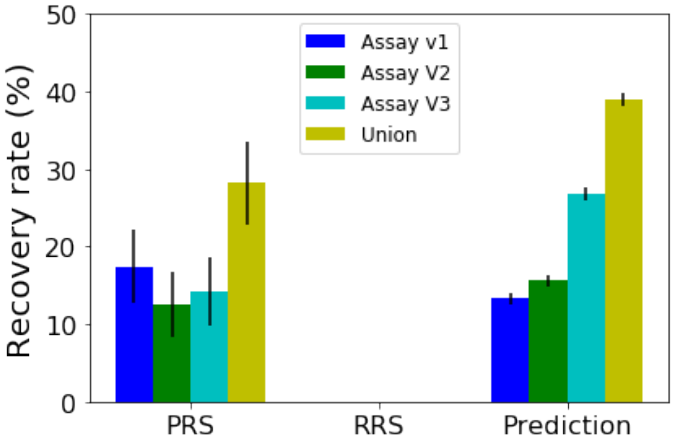

Supplementary Figure 9. **Recovery rate of different prediction methods.** PRS: a set of high confidence binary PPIs curated from the published literature. RRS: a set of pairs selected at random from the search space.

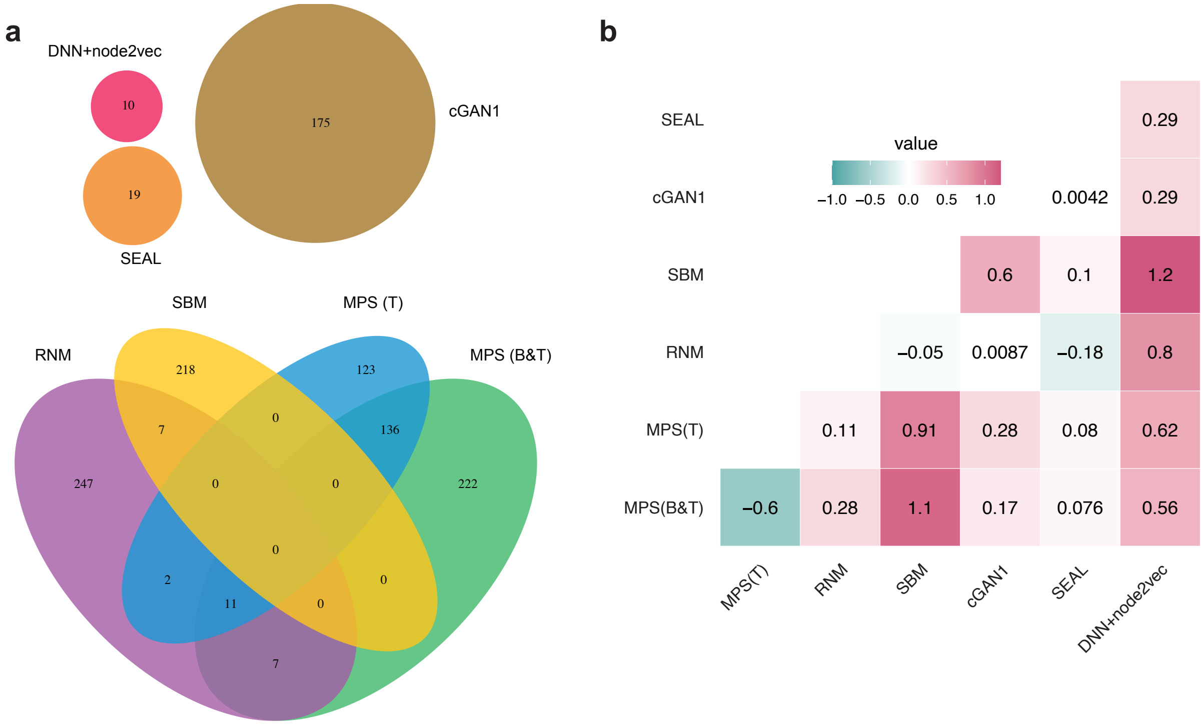

Supplementary Figure 10. **Positive PPIs predicted by the top-seven methods do not overlap and represent different areas in the original interactome. a:** Venn diagram for the overlap between the positive PPIs predicted by the top-seven method. **b:** Network separation of proteins involved in the positive PPIs predicted by top-seven methods. The network separation [81] between two sets of proteins is greater than or equal to 0 (red colored cells) if the two protein sets are well separated, if they are overlapping, the separation has a negative value (green colored cells).

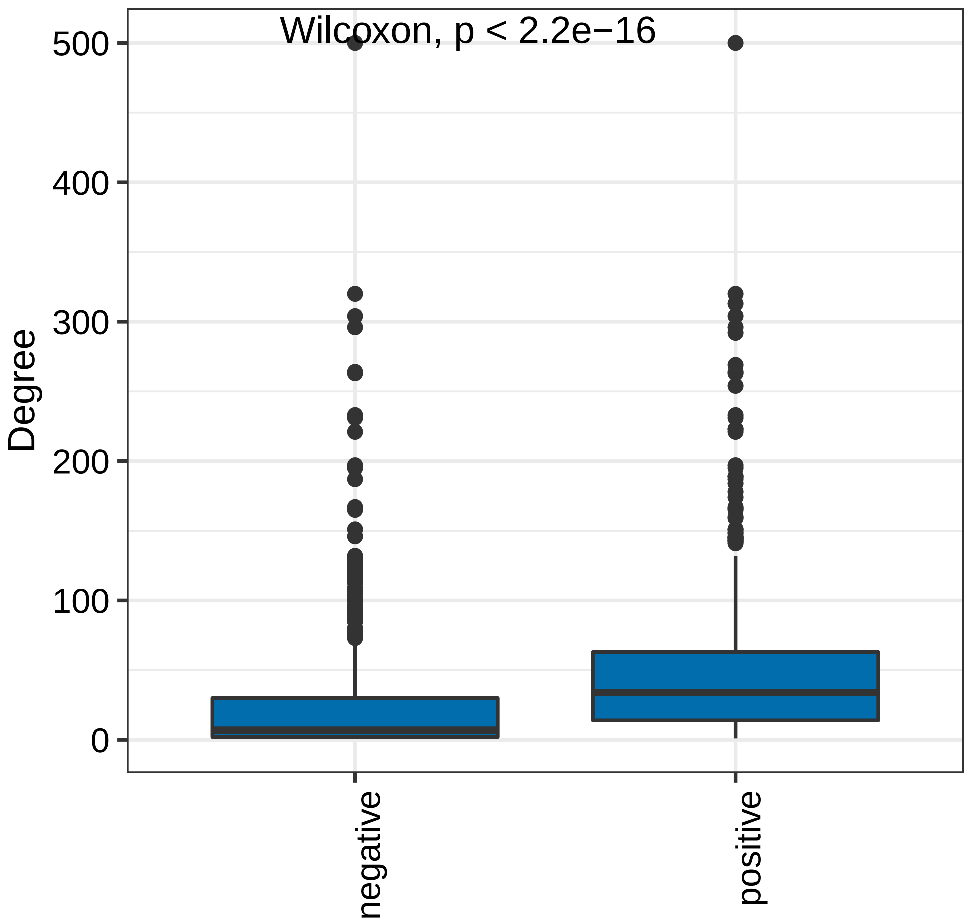

Supplementary Figure 11. **Degree distribution of proteins involved in experimentally validated PPIs.** We compared the degree of proteins (in original HuRI) involved in experimentally validated positive and negative PPIs. P-value was calculated using Wilcoxon test.

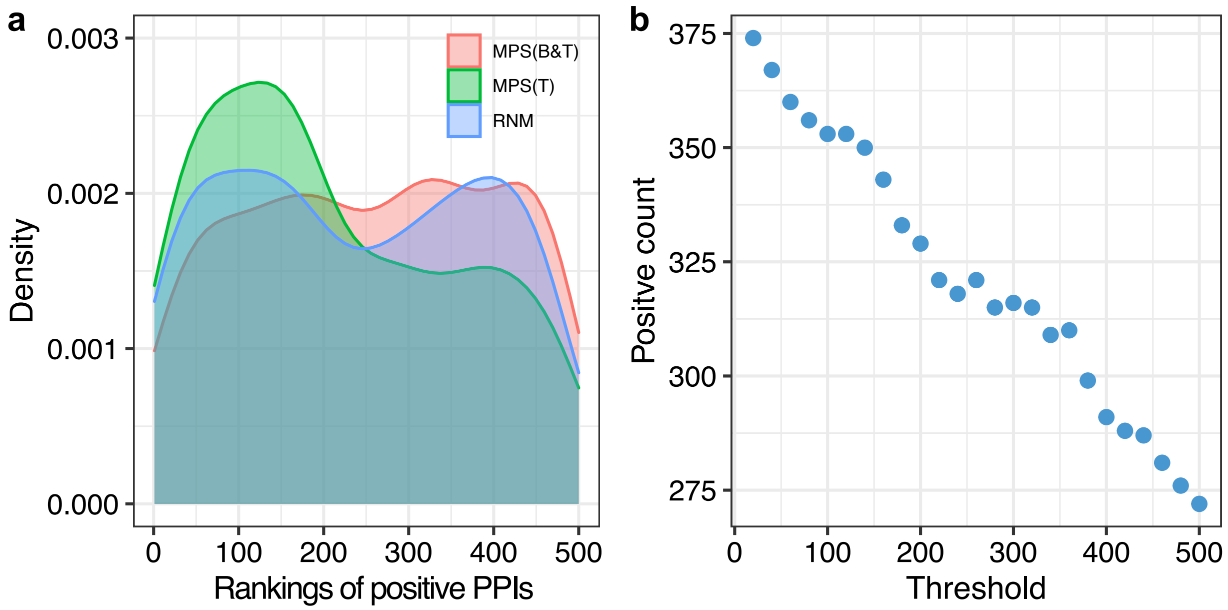

Supplementary Figure 12. **Combining the top-500 predicted previously uncharacterized PPIs from top-three methods. a:** The ranking distributions (kernel density estimate) of those positive PPIs validated experimentally for each method: MPS(B&T), MPS(T) and RNM. **b**: Number of positive PPIs after combining MPS(B&T), MPS(T) and RNM together: we combined the top-N_k PPIs predicted by MPS(B&T) and top-$[(500 - N_k)/2]$ from MPS(T) and RNM where $0 \leq N_k \leq 500$ is a parameter used to tune the ratio of PPIs predicted by different methods. Note that during the combining process, we ensured that those PPIs predicted by multiple methods appear only once in the top-500 PPI list.

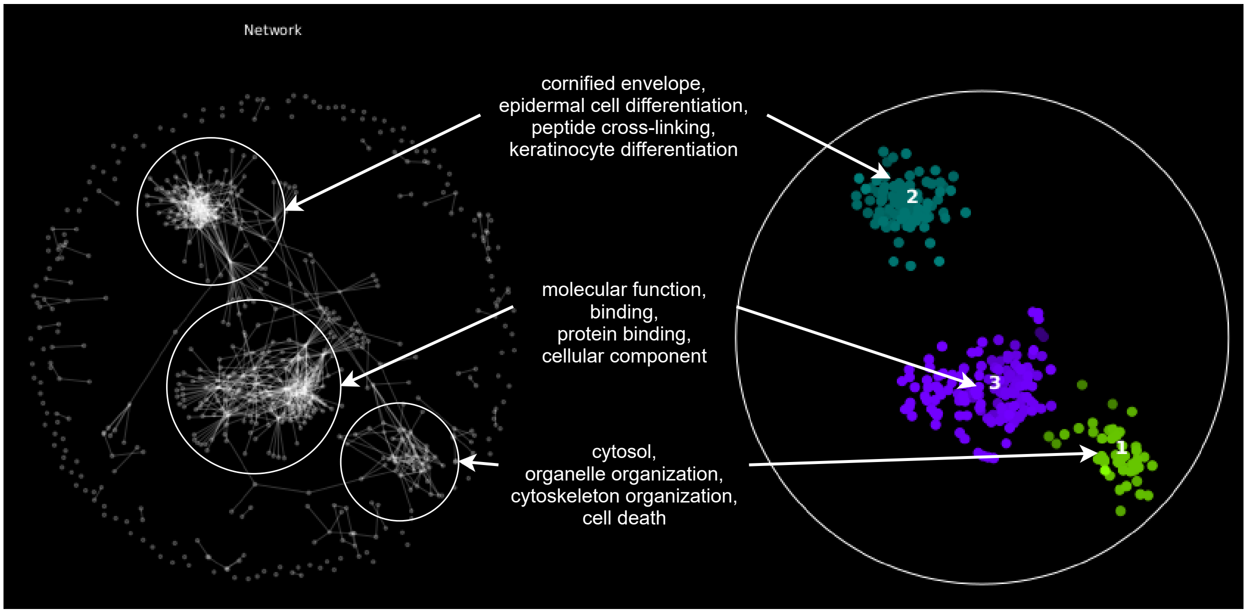

Supplementary Figure 13. **Functional relationships among the previously uncharacterized human PPIs discovered in this project.** Left: This network consists of all 1,177 previously uncharacterized human PPIs predicted by the top-seven methods and validated by Y2H assays. Right: functional modules discovered by SAFE [82]. Gene Ontology [83] (GO) terms for each gene were extracted from FuncAssociate [84]. The neighborhood radius is set to be 0.15 in SAFE. Note that the PPIs in the first and the second modules are mostly predicted by MPS(B&T) and MPS(T) but the rest of the PPIs are predicted by the other methods.

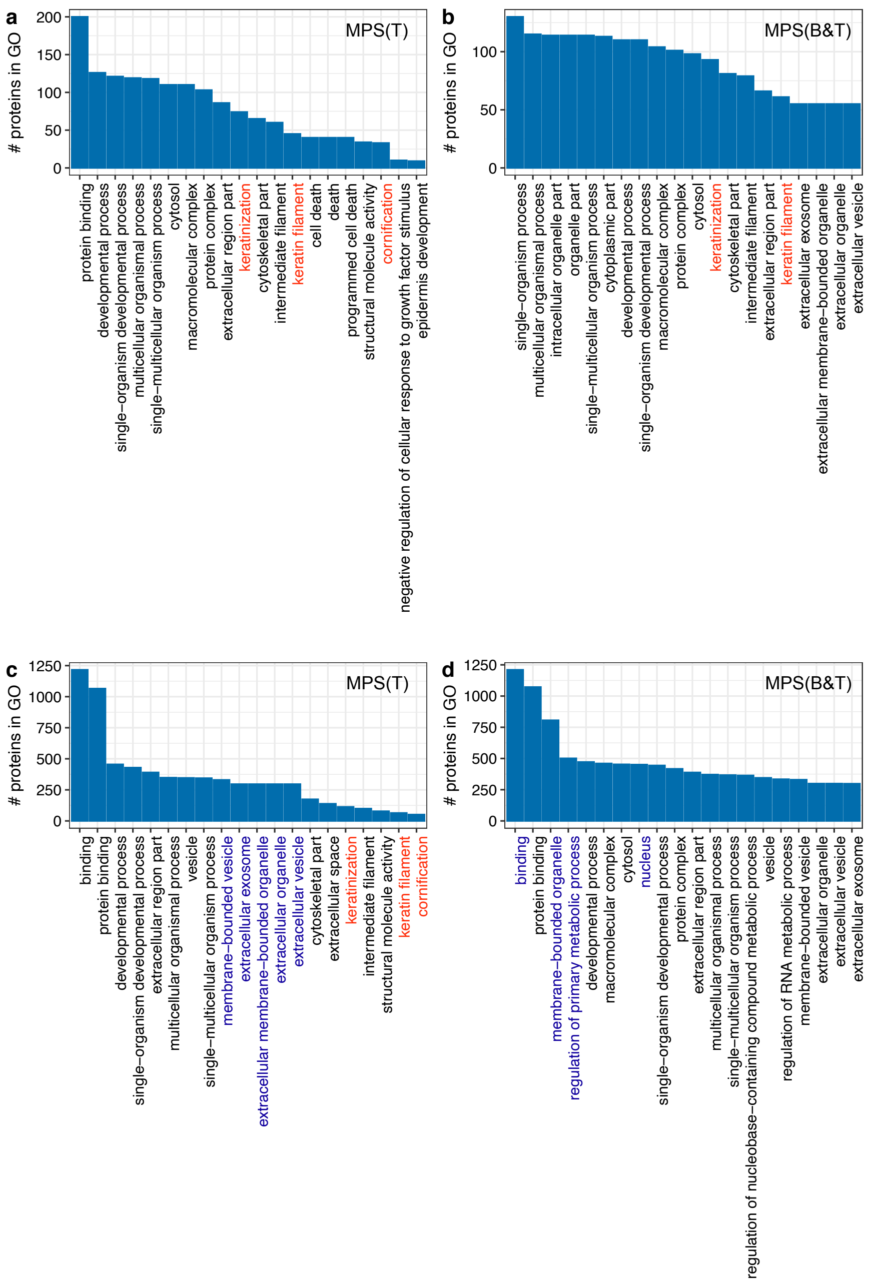

Supplementary Figure 14. **Functional analysis of proteins involved in the predicted PPIs.** We firstly extracted the Gene Ontology (GO) terms for those proteins involved in the PPIs predicted by MPS(T) and MPS(B&T) using FuncAssociate [84]. Then, we showed the number of proteins associated with each GO term. **a-b,** Top-20 GO terms associated with proteins involved in the top-500 PPIs predicted by MPS(T) (a) and MPS(B&T) (b). **c-d**, Top-20 GO terms associated with proteins involved in the top-5,000 PPIs predicted by MPS(T) (c) and MPS(B&T) (d).

[1] J. De Las Rivas and C. Fontanillo, Protein–protein interactions essentials: key concepts to building and analyzing interactome networks, PLoS Comput Biol **6**, e1000807 (2010).

[2] K. Titeca, I. Lemmens, J. Tavernier, and S. Eyckerman, Discovering cellular protein-protein interactions: Technological strategies and opportunities, Mass Spectrometry Reviews **38**, 79 (2019).

[3] A. R. Mashaghi, A. Ramezanpour, and V. Karimipour, Investigation of a protein complex network, The European Physical Journal B-Condensed Matter and Complex Systems **41**, 113 (2004).

[4] M. Al Hasan, V. Chaoji, S. Salem, and M. Zaki, Link prediction using supervised learning, in *SDM06: Workshop on link analysis, counter-terrorism and security* (2006).

[5] R. R. Sarukkai, Link prediction and path analysis using markov chains, Computer Networks **33**, 377 (2000).

[6] L. Lü, C.-H. Jin, and T. Zhou, Similarity index based on local paths for link prediction of complex networks, Physical Review E **80**, 046122 (2009).

[7] W. Liu and L. Lü, Link prediction based on local random walk, EPL (Europhysics Letters) **89**, 58007 (2010).

[8] D. Liben-Nowell and J. Kleinberg, The link-prediction problem for social networks, Journal of the Association for Information Science and Technology **58**, 1019 (2007).

[9] R. N. Lichtenwalter, J. T. Lussier, and N. V. Chawla, New perspectives and methods in link prediction, in *Proceedings of the 16th ACM SIGKDD International conference on Knowledge discovery and data mining* (ACM, 2010) pp. 243–252.

[10] B. Barzel and A.-L. Barabási, Network link prediction by global silencing of indirect correlations, Nature Biotechnology **31**, 720 (2013).

[11] P. Gainza, F. Sverrisson, F. Monti, E. Rodola, D. Boscaini, M. Bronstein, and B. Correia, Deciphering interaction fingerprints from protein molecular surfaces using geometric deep learning, Nature Methods **17**, 184 (2020).

[12] T. Zhou, Z. Kuscsik, J.-G. Liu, M. Medo, J. R. Wakeling, and Y.-C. Zhang, Solving the apparent diversity-accuracy dilemma of recommender systems, Proceedings of the National Academy of Sciences of the United States of America **107**, 4511 (2010).

[13] L. A. Adamic and E. Adar, Friends and neighbors on the web, Social Networks **25**, 211 (2003).

[14] P. Jaccard, Étude comparative de la distribution florale dans une portion des alpes et des jura, Bull Soc Vaudoise Sci Nat **37**, 547 (1901).

[15] A.-L. Barabási and R. Albert, Emergence of scaling in random networks, Science **286**, 509 (1999).

[16] T. Zhou, L. Lü, and Y.-C. Zhang, Predicting missing links via local information, The European Physical Journal B-Condensed Matter and Complex Systems **71**, 623 (2009).

[17] Q. Ou, Y.-D. Jin, T. Zhou, B.-H. Wang, and B.-Q. Yin, Power-law strength-degree correlation from resource-allocation dynamics on weighted networks, Physical Review E **75**, 021102 (2007).

[18] E. Ravasz, A. L. Somera, D. A. Mongru, Z. N. Oltvai, and A.-L. Barabási, Hierarchical organization of modularity in metabolic networks, Science **297**, 1551 (2002).

[19] T. A. Sorensen, A method of establishing groups of equal amplitude in plant sociology based on similarity of species content and its application to analyses of the vegetation on danish commons, Biol. Skar. **5**, 1 (1948).

[20] I. A. Kovács, K. Luck, K. Spirohn, Y. Wang, C. Pollis, S. Schlabach, W. Bian, D.-K. Kim, N. Kishore, T. Hao, *et al.*, Network-based prediction of protein interactions, Nature Communications **10**, 1 (2019).

[21] Y. Chen, W. Wang, J. Liu, J. Feng, and X. Gong, Protein interface complementarity and gene duplication improve link prediction of protein-protein interaction network, Frontiers in Genetics **11** (2020).

[22] L. Katz, A new status index derived from sociometric analysis, Psychometrika **18**, 39 (1953).

[23] L. Lü, L. Pan, T. Zhou, Y.-C. Zhang, and H. E. Stanley, Toward link predictability of complex networks, Proceedings of the National Academy of Sciences of the United States of America **112**, 2325 (2015).

[24] S. Pitre, F. Dehne, A. Chan, J. Cheetham, A. Duong, A. Emili, M. Gebbia, J. Greenblatt, M. Jessulat, N. Krogan, *et al.*, Pipe: a protein-protein interaction prediction engine based on the re-occurring short polypeptide sequences between known interacting protein pairs, BMC Bioinformatics **7**, 1 (2006).

[25] L. Becchetti, A. Fazzone, and L. Martini, Network and sequence-based prediction of protein-protein interactions, ArXiv preprint arXiv:2107.03694 (2021).

[26] M. Kitsak, Latent geometry for complementarity-driven networks, arXiv preprint arXiv:2003.06665 (2020).

[27] E. A. Leicht, P. Holme, and M. E. Newman, Vertex similarity in networks, Physical Review E **73**, 026120 (2006).

[28] Y. Dong, Q. Ke, B. Wang, and B. Wu, Link prediction based on local information, in *2011 International Conference on Advances in Social Networks Analysis and Mining* (IEEE, 2011) pp. 382–386.

[29] F. Tan, Y. Xia, and B. Zhu, Link prediction in complex networks: a mutual information perspective, PloS One **9**, e107056 (2014).

[30] C. V. Cannistraci, G. Alanis-Lobato, and T. Ravasi, From link-prediction in brain connectomes and protein interactomes to the local-community-paradigm in complex networks, Scientific reports **3**, 1 (2013).

[31] V. Martínez, F. Berzal, and J.-C. Cubero, A survey of link prediction in complex networks, ACM Computing Surveys (CSUR) **49**, 1 (2016).

[32] A. Kumar, S. S. Singh, K. Singh, and B. Biswas, Link prediction techniques, applications, and performance: A survey, Physica A: Statistical Mechanics and its Applications **553**, 124289 (2020).

[33] T. Zhou, Progresses and challenges in link prediction, ArXiv preprint arXiv:2102.11472 (2021).

[34] M. Zhang and Y. Chen, Link prediction based on graph neural networks, in *NeurIPS* (2018).

[35] H. C. White, S. A. Boorman, and R. L. Breiger, Social structure from multiple networks. i. blockmodels of roles and positions, American Journal of Sociology **81**, 730 (1976).

[36] P. W. Holland, K. B. Laskey, and S. Leinhardt, Stochastic blockmodels: First steps, Social Networks **5**, 109 (1983).

[37] R. Guimerà and M. Sales-Pardo, Missing and spurious interactions and the reconstruction of complex networks, Proceedings of the National Academy of Sciences of the United States of America **106**, 22073 (2009).

[38] A. Clauset, C. Moore, and M. E. Newman, Hierarchical structure and the prediction of missing links in networks, Nature **453**, 98 (2008).

[39] S. Colonnese, P. Di Lorenzo, T. Cattai, G. Scarano, and F. D. V. Fallani, A joint markov model for communities, connectivity and signals defined over graphs, IEEE Signal Processing Letters **27**, 1160 (2020).

[40] N. Tremblay and P. Borgnat, Graph wavelets for multiscale community mining, IEEE Transactions on Signal Processing **62**, 5227 (2014).

[41] S. Colonnese, M. Petti, L. Farina, G. Scarano, and F. Cuomo, Protein-protein interaction prediction via graph signal processing, IEEE Access **9**, 142681 (2021).

[42] V. Martínez, F. Berzal, and J.-C. Cubero, A survey of link prediction in complex networks, ACM Computing Surveys (CSUR) **49**, 69 (2017).

[43] N. Friedman, L. Getoor, D. Koller, and A. Pfeffer, Learning probabilistic relational models, in *IJCAI*, Vol. 99 (1999) pp. 1300–1309.

[44] D. Heckerman, C. Meek, and D. Koller, Probabilistic entity-relationship models, prms, and plate models, Introduction to Statistical Relational Learning , 201 (2007).

[45] K. Yu, W. Chu, S. Yu, V. Tresp, and Z. Xu, Stochastic relational models for discriminative link prediction, in *Advances in neural information processing systems* (2007) pp. 1553–1560.

[46] C. H. Ding, T. Li, and M. I. Jordan, Convex and semi-nonnegative matrix factorizations, IEEE transactions on pattern analysis and machine intelligence **32**, 45 (2008).

[47] R. Pech, D. Hao, L. Pan, H. Cheng, and T. Zhou, Link prediction via matrix completion, EPL (Europhysics Letters) **117**, 38002 (2017).

[48] L. Torres, K. S. Chan, and T. Eliassi-Rad, Glee: Geometric laplacian eigenmap embedding, Journal of Complex Networks **8**, cnaa007 (2020).

[49] T. Mikolov, K. Chen, G. Corrado, and J. Dean, Efficient estimation of word representations in vector space, arXiv preprint arXiv:1301.3781 (2013).

[50] H. Tong, C. Faloutsos, and J.-Y. Pan, Fast random walk with restart and its applications, in:Proceedings of the 6th International Conference on Data Mining (2006).

[51] G. Jeh and J. Widom, Simrank: a measure of structural-context similarity, in *Proceedings of the eighth ACM SIGKDD international conference on Knowledge discovery and data mining* (ACM, 2002) pp. 538–543.

[52] B. Perozzi, R. Al-Rfou, and S. Skiena, Deepwalk: Online learning of social representations, in *Proceedings of the 20th ACM SIGKDD international conference on Knowledge discovery and data mining* (ACM, 2014) pp. 701–710.

[53] T. Mikolov, I. Sutskever, K. Chen, G. S. Corrado, and J. Dean, Distributed representations of words and phrases and their compositionality, in *Advances in neural information processing systems* (2013) pp. 3111–3119.

[54] J. Tang, M. Qu, M. Wang, M. Zhang, J. Yan, and Q. Mei, Line: Large-scale information network embedding, in *Proceedings of the 24th International Conference on World Wide Web* (International World Wide Web Conferences Steering Committee, 2015) pp. 1067–1077.

[55] A. Grover and J. Leskovec, node2vec: Scalable feature learning for networks, in *Proceedings of the 22nd ACM SIGKDD international conference on Knowledge discovery and data mining* (2016) pp. 855–864.

[56] L. Yang, J.-F. Xia, and J. Gui, Prediction of protein-protein interactions from protein sequence using local descriptors, Protein and Peptide Letters **17**, 1085 (2010).

[57] Y. Guo, L. Yu, Z. Wen, and M. Li, Using support vector machine combined with auto covariance to predict protein–protein interactions from protein sequences, Nucleic Acids Research **36**, 3025 (2008).

[58] Y. Z. Zhou, Y. Gao, and Y. Y. Zheng, Prediction of protein-protein interactions using local description of amino acid sequence, in *Advances in Computer Science and Education Applications* (Springer, 2011) pp. 254–262.

[59] Z.-H. You, L. Zhu, C.-H. Zheng, H.-J. Yu, S.-P. Deng, and Z. Ji, Prediction of protein-protein interactions from amino acid sequences using a novel multi-scale continuous and discontinuous feature set, in *BMC Bioinformatics*, Vol. 15 (Springer, 2014) pp. 1–9.

[60] B. Yu, C. Chen, Z. Yu, A. Ma, B. Liu, and Q. Ma, Prediction of protein-protein interactions based on elastic net and deep forest, BioRxiv (2020).

[61] M. Kong, Y. Zhang, D. Xu, W. Chen, and M. Dehmer, Fctp-wsrc: protein–protein interactions prediction via weighted sparse representation based classification, Frontiers in Genetics **11**, 18 (2020).

[62] Z.-H. You, Y.-K. Lei, L. Zhu, J. Xia, and B. Wang, Prediction of protein-protein interactions from amino acid sequences with ensemble extreme learning machines and principal component analysis, in *BMC Bioinformatics*, Vol. 14 (Springer, 2013) pp. 1–11.

[63] X. Du, S. Sun, C. Hu, Y. Yao, Y. Yan, and Y. Zhang, Deepppi: boosting prediction of protein–protein interactions with deep neural networks, Journal of Chemical Information and Modeling **57**, 1499 (2017).

[64] Y. Guo and X. Chen, A deep learning framework for improving protein interaction prediction using sequence properties, BioRxiv , 843755 (2019).

[65] S. Hashemifar, B. Neyshabur, A. A. Khan, and J. Xu, Predicting protein–protein interactions through sequence-based deep learning, Bioinformatics **34**, i802 (2018).

[66] X.-W. Wang, Y. Chen, and Y.-Y. Liu, Link prediction through deep generative model, Iscience **23**, 101626 (2020).

[67] O. M. Balogh, B. Benczik, A. Horváth, M. Pétervári, P. Csermely, P. Ferdinandy, and B. Ágg, Efficient link prediction in the protein–protein interaction network using topological information in a generative adversarial network machine learning model, **23**, 78.

[68] I. Gulrajani, F. Ahmed, M. Arjovsky, V. Dumoulin, and A. C. Courville, Improved training of wasserstein gans, in *Advances in Neural Information Processing Systems* (2017) pp. 5769–5779.

[69] M. Arjovsky, S. Chintala, and L. Bottou, Wasserstein generative adversarial networks, in *International conference on machine learning* (PMLR, 2017) pp. 214–223.

[70] P. Isola, J.-Y. Zhu, T. Zhou, and A. A. Efros, Image-to-image translation with conditional adversarial networks, in *Proceedings of the IEEE conference on computer vision and pattern recognition* (2017) pp. 1125–1134.

[71] W. L. Hamilton, R. Ying, and J. Leskovec, Inductive representation learning on large graphs, arXiv preprint arXiv:1706.02216 (2017).

[72] A. Morgat, T. Lombardot, E. Coudert, K. Axelsen, T. B. Neto, S. Gehant, P. Bansal, J. Bolleman, E. Gasteiger, E. De Castro, *et al.*, Enzyme annotation in uniprotkb using rhea, Bioinformatics **36**, 1896 (2020).

[73] T. Yamamoto, Crystal graph neural networks for data mining in materials science (2019).

[74] K. Huang, C. Xiao, L. M. Glass, M. Zitnik, and J. Sun, Skipgnn: predicting molecular interactions with skip-graph networks, Scientific Reports **10**, 1 (2020).

[75] P. Shannon, A. Markiel, O. Ozier, N. S. Baliga, J. T. Wang, D. Ramage, N. Amin, B. Schwikowski, and T. Ideker, Cytoscape: a software environment for integrated models of biomolecular interaction networks, Genome Research **13**, 2498 (2003).

[76] X. Zeng, L. Liu, L. Lü, and Q. Zou, Prediction of potential disease-associated micrornas using structural perturbation method, Bioinformatics **34**, 2425 (2018).

[77] A. Vázquez, A. Flammini, A. Maritan, and A. Vespignani, Modeling of protein interaction networks, Complexus **1**, 38 (2003).

[78] M. Zitnik, J. Leskovec, *et al.*, Prioritizing network communities, Nature communications **9**, 1 (2018).

[79] B. Reilly, Social choice in the south seas: Electoral innovation and the borda count in the pacific island countries, International Political Science Review **23**, 355 (2002).

[80] G. Csardi, T. Nepusz, *et al.*, The igraph software package for complex network research, InterJournal, complex systems **1695**, 1 (2006).

[81] J. Menche, A. Sharma, M. Kitsak, S. D. Ghiassian, M. Vidal, J. Loscalzo, and A.-L. Barabási, Uncovering disease-disease relationships through the incomplete interactome, Science **347** (2015).

[82] A. Baryshnikova, Systematic functional annotation and visualization of biological networks, Cell Systems **2**, 412 (2016).

[83] M. Ashburner, C. A. Ball, J. A. Blake, D. Botstein, H. Butler, J. M. Cherry, A. P. Davis, K. Dolinski, S. S. Dwight, J. T. Eppig, *et al.*, Gene ontology: tool for the unification of biology, Nature Genetics **25**, 25 (2000).

[84] G. F. Berriz, O. D. King, B. Bryant, C. Sander, and F. P. Roth, Characterizing gene sets with funcassociate, Bioinformatics **19**, 2502 (2003).
