# Supplementary material for: Assessment of community efforts to advance network-based prediction of protein–protein interactions
Source: Nat Commun. 2023 Mar 22;14:1582. doi: 10.1038/s41467-023-37079-7 (PMC10033937; doi:10.1038/s41467-023-37079-7)
Supplement: Supplementary file 2 — Reporting Summary [file 41467_2023_37079_MOESM2_ESM.pdf]

nature portfolio

Corresponding author(s): Yang-Yu Liu
Last updated by author(s): Feb 28, 2023

Reporting Summary

Nature Portfolio wishes to improve the reproducibility of the work that we publish. This form provides structure for consistency and transparency in reporting. For further information on Nature Portfolio policies, see our Editorial Policies and the Editorial Policy Checklist.

Statistics

For all statistical analyses, confirm that the following items are present in the figure legend, table legend, main text, or Methods section.

n/a	Confirmed	
☐	☒	The exact sample size (n) for each experimental group/condition, given as a discrete number and unit of measurement
☒	☐	A statement on whether measurements were taken from distinct samples or whether the same sample was measured repeatedly
☐	☒	The statistical test(s) used AND whether they are one- or two-sided<br>*Only common tests should be described solely by name; describe more complex techniques in the Methods section.*
☒	☐	A description of all covariates tested
☒	☐	A description of any assumptions or corrections, such as tests of normality and adjustment for multiple comparisons
☐	☒	A full description of the statistical parameters including central tendency (e.g. means) or other basic estimates (e.g. regression coefficient) AND variation (e.g. standard deviation) or associated estimates of uncertainty (e.g. confidence intervals)
☐	☒	For null hypothesis testing, the test statistic (e.g. F, t, r) with confidence intervals, effect sizes, degrees of freedom and P value noted<br>*Give P values as exact values whenever suitable.*
☒	☐	For Bayesian analysis, information on the choice of priors and Markov chain Monte Carlo settings
☐	☒	For hierarchical and complex designs, identification of the appropriate level for tests and full reporting of outcomes
☐	☒	Estimates of effect sizes (e.g. Cohen's d, Pearson's r), indicating how they were calculated

Our web collection on statistics for biologists contains articles on many of the points above.

Software and code

Policy information about availability of computer code

Data collection	BioGRID dataset was collected using R package: rTRM.
Data analysis	R 4.0.2 was used to visualized the results. Network was visualized using Gephi (version 0.9). Ranking algorithms were implemented using rankaggretation package (https://github.com/djcunningham0/rankaggregation). Matlab code for predictability was downloaded from: https://github.com/lecea/SPM-code.git.<br><br>Code availability: all other custom Python and Matlab prediction codes and public available codes can be found at: https://github.com/spxuw/PPI-Prediction-Project.

For manuscripts utilizing custom algorithms or software that are central to the research but not yet described in published literature, software must be made available to editors and reviewers. We strongly encourage code deposition in a community repository (e.g. GitHub). See the Nature Portfolio guidelines for submitting code & software for further information.

Data

Policy information about availability of data

All manuscripts must include a data availability statement. This statement should provide the following information, where applicable:

- Accession codes, unique identifiers, or web links for publicly available datasets
- A description of any restrictions on data availability
- For clinical datasets or third party data, please ensure that the statement adheres to our policy

All data are available at https://github.com/spxuw/PPI-Prediction-Project. Source data are provided with this paper.

Human research participants

Policy information about studies involving human research participants and Sex and Gender in Research.

Reporting on sex and gender	N/A
Population characteristics	N/A
Recruitment	N/A
Ethics oversight	N/A

Note that full information on the approval of the study protocol must also be provided in the manuscript.

Field-specific reporting

Please select the one below that is the best fit for your research. If you are not sure, read the appropriate sections before making your selection.

☒ Life sciences ☐ Behavioural & social sciences ☐ Ecological, evolutionary & environmental sciences

For a reference copy of the document with all sections, see nature.com/documents/nr-reporting-summary-flat.pdf

Life sciences study design

All studies must disclose on these points even when the disclosure is negative.

Sample size	The top-500 PPIs from top-seven methods, in total 3,276 predicted PPIs were experimentally validated. In the computational validation, we found that the ranking of those top-ranking methods is consistent using precision at 500, 2000 and 5000. Therefore, we validated top-500 PPIs, which is experimentally affordable.
Data exclusions	No data were excluded.
Replication	We used the Y2H assay which has been used in previous study (10.1038/s41586-020-2188-x), showing that we have significantly higher correlation between replicates than that between samples.
Randomization	Screen paris and controls for Y2H pairwise test, MAPPIT and GPCA were randomly arranged on well plates.
Blinding	Manual scoring of Y2H pairwise tests was blinded.

Reporting for specific materials, systems and methods

We require information from authors about some types of materials, experimental systems and methods used in many studies. Here, indicate whether each material, system or method listed is relevant to your study. If you are not sure if a list item applies to your research, read the appropriate section before selecting a response.

Materials & experimental systems

n/a	Involved in the study
☒	☐ Antibodies
☐	☒ Eukaryotic cell lines
☒	☐ Palaeontology and archaeology
☒	☐ Animals and other organisms
☒	☐ Clinical data
☒	☐ Dual use research of concern

Methods

n/a	Involved in the study
☒	☐ ChIP-seq
☒	☐ Flow cytometry
☒	☐ MRI-based neuroimaging

Eukaryotic cell lines

Policy information about cell lines and Sex and Gender in Research

Cell line source(s)	Yeast strains - Y8800 and Y8930: the same strain used in previous study (Luck, et al. Nature 580, 402–408 (2020))
Authentication	Not re-authenticated.
Mycoplasma contamination	All the cell lines were tested with DNA strain with mycoplasma contamination and free of contamination.
Commonly misidentified lines (See ICLAC register)	No commonly misidentified lines were used.
